# Green technologies for extracting plant waste functional ingredients and new food formulation: A review

**DOI:** 10.1111/1750-3841.17487

**Published:** 2024-11-04

**Authors:** Giulia Basile, Lucia De Luca, Giovanni Sorrentino, Martina Calabrese, Mariarca Esposito, Fabiana Pizzolongo, Raffaele Romano

**Affiliations:** ^1^ Department of Agricultural Sciences University of Naples Federico II, Piazza Carlo di Borbone I Portici (NA) Italy

**Keywords:** bioactive compounds, functional foods, green extraction, polyphenols, recovery waste

## Abstract

Nowadays, there is a growing interest in food waste recovery by both consumers and companies. Food waste of plant origin is a source of bioactive compounds, such as phenolic acids, anthocyanins, flavonoids, phytosterols, carotenoids, and tocopherols, with well‐known antioxidant, anti‐glycemic, and antimicrobial properties. The use of green and sustainable technologies to recover bioactive compounds from food waste is a possible solution to valorize waste following the principles of green chemistry. Furthermore, today's consumers are more attracted, informed, and aware of the benefits associated with the consumption of functional foods, and with this in mind, the use of extracts rich in beneficial compounds obtained by green technologies from food waste can be a valid alternative to prepare functional foods. In this review, the recovery of polyphenols and fibers with green technologies from food waste for the formulation of functional foods was presented.

## 1 INTRODUCTION

Nowadays, it is necessary to adopt approaches aimed at converting the existing linear and unsustainable agrifood chain into a circular and more efficient production and consumption system to address the present and future requirements of the human population (Othman et al., 2022). In order to mitigate the enduring adverse economic, social, and environmental outcomes of ongoing waste generation, the European Commission has embraced a framework for advancing a circular economy (Georgiev et al., 2022).

Food processing inevitably generates byproducts (Vojvodić Cebin et al., 2020), and vegetable food wastes are rich in a lot of bioactive compounds, such as polyphenols, carotenoids, lignins, fiber, tannins, alkaloids, sterols, triterpenes, peptides, and carbohydrates. These compounds exhibit a lot of beneficent activities, including antioxidant, antibacterial, anti‐diabetic, anticancer, antihypertensive, anti‐inflammatory, anticholesterol, and protective effects on the cardiocirculatory system (Doria et al., 2021).

Therefore, consumers’ growing understanding of the relationship between diet and health stimulates opportunities for new foods with health benefits (Aiello et al., 2020; Sun‐Waterhouse & Wadhwa, 2013). Consequently, the possibility of recovering extracts rich in bioactive compounds represents an excellent opportunity to coincide with the production of new foods with functional properties, the implementation of new techniques that follow the principles of green chemistry, and the valorization of food waste.

To align with the principles of the green economy, bioactive compounds from plant food waste should be extracted using environmentally friendly, sustainable, and cost‐effective methods (Panzella et al., 2020). Based on this premise, this review aims to outline the process of recovering bioactive compounds from plant food waste using green technologies and explore their potential utilization as ingredients in the development of functional foods.

## 2 POLYPHENOLS IN FOOD WASTE

Polyphenols are phytochemical compounds associated with benefits, particularly in relation to lifestyle diseases and oxidative stress (Mansour et al., 2018). Consequently, they are crucial for human well‐being, as a deficiency in antioxidant molecules can lead to potential health issues (Deng et al., 2011). They have different properties, such as anti‐inflammatory, antiviral, and antimicrobial, along with antioxidant capacity (Ignat et al., 2011).

Among the various benefits related to the consumption of polyphenols is the inhibition of α‐amylase and/or α‐glucosidase enzymes, which helps modulate the body's glycemic response to carbohydrates. This mechanism is influenced by the type, structure, and concentration of polyphenols. For instance, monomeric polyphenols can deactivate digestive enzymes by blocking their catalytic sites, whereas polymeric polyphenols can form nondigestible complexes with digestive enzymes, slowing down carbohydrate digestion and reducing the glycemic peak (Kan et al., 2020).

Moreover, during carbohydrate digestion, polyphenols can exert hypoglycemic effects by inhibiting salivary and pancreatic α‐amylase and α‐glucosidase enzymes at the small intestinal brush border. They also inhibit glucose absorption, stimulate insulin secretion, and protect pancreatic β‐cells from glucotoxicity. Additionally, polyphenols can suppress glucose release from the liver and enhance glucose uptake in peripheral tissues (Kim et al., 2016).

Polyphenols have shown additional benefits for intestinal health by regulating intestinal flora, alleviating inflammation, and potentially preventing the progression of chronic diseases such as tumors (Li et al., 2021). Various in vivo and in vitro studies have highlighted the favorable effects of specific polyphenols, such as punicalagin and ellagic acid, which include improving insulin sensitivity, inhibiting α‐glucosidase, and reducing oxidative stress and lipid levels (Banihani et al., 2013).

Plant wastes represent a valuable source of polyphenols that should be recovered, and the extraction of these compounds from food waste poses a critical challenge for sustainable industrial processes (Barbera, 2020).

The citrus peels are rich in flavonoids and phenolic acid. Gómez‐Mejía et al. (2019) evaluated the polyphenol extraction from clementine, lemon, and orange peels, reporting that the most abundant molecules found are hesperidin (280–673 mg/g dry weight), rutin (3.3–4.7 mg/g dry weight), *p*‐coumaric acid (∼1.4 mg/g extract), and *trans‐*ferulic acid (∼1.4 mg/g extract). The most concentrated compound was hesperidin. Tabeshpour et al. (2020) reported that this compound can protect against liver damage from inflammation and/or oxidative stress mediated by natural and chemical toxins, such as lipopolysaccharide, concanavalin A, microcystin, and ethanol.

Moreover, the apple pomace, the main byproduct obtained after crushing and pressing apples for juice production, is an important source of flavonoids, phloridzin, and phenolic acid. In this way, Wu Li et al. (2020) evaluated the extraction of polyphenols from apple pomace, showing that the major polyphenols found are quercetin‐3‐*O*‐glucoside (0–51.73 mg/kg dry weight), quercetin‐3‐*O*‐galactoside (0–65.54 mg/kg dry weight), phloridzin (0–35.28 mg/kg dry weight), quercetin‐3‐*O*‐rhamnoside (0–37.16 mg/kg dry weight), protocatechuic acid (0–30.50 mg/kg dry weight), and chlorogenic acid (0–160.40 mg/kg dry weight). Phenolic compounds found in apples exhibit potent radical‐scavenging activity. Specifically, phloridzin, a compound present in apples, can competitively inhibit glucose transport by binding to the glucose moiety of the Na^+^/glucose co‐transporter. This action is recognized as having anti‐diabetic properties, making phloridzin a potential anti‐diabetic agent. (Lavelli & Corti, 2011).

Grape pomace, a byproduct of wine making consisting mainly of grape skin residues, broken cells with pulp remains, stalks, and seeds (Ruggieri et al., 2009), is rich in anthocyanins, catechins, and flavonols that offer numerous health benefits due to their antioxidant, anticancer, antifungal, and antibacterial properties (Chedea et al., 2018). Dimou and Koutelidakis (2016) reported that polyphenols in grape pomace, such as malvidin 3,5‐diglucoside and malvidin glucosides, may serve as potent inhibitors of lipid peroxidation of human low‐density lipoproteins (LDLs). Catechin and epicatechin, also present in grape pomace, are known for their antioxidant properties. Proanthocyanidins found in grape seeds can prevent inflammatory conditions by scavenging free radicals, inhibiting lipid peroxidation, and blocking the formation of pro‐inflammatory cytokines.

Finally, tomato peel is a source of lycopene and polyphenols (George et al., 2004; Toor & Savage, 2005), whereas the tomato seeds have been shown to contain about 20% oil of high nutritional quality (Eller et al., 2010), characterized by the high amount of oleic acid (27.16/100 g of total fatty acid) and linoleic acid (48.69 g/100 g of total fatty acid) (Li et al., 2020).

## 3 FIBER RECOVERY BY FOOD WASTE

Dietary fibers are plant constituents that escape digestion and absorption in the upper human intestine but can be partially or completely fermented in the lower intestine. They are an important diet component of humans with recognized health properties that can reduce the risk of specific chronic diseases such as Type 2 diabetes and coronary heart disease (Yegin et al., 2020). It has been proven that a diet rich in β‐glucan can improve immune system function and provide protection against hypertension, stroke, cardiovascular disease, and Type 2 diabetes (Koksel et al., 2024). Xyloglucan is a polysaccharide consisting of a cellulose polymer branched with d‐xylose linked by 1 → 6 bonds. Xylose residues at position O6 can further bind with galactose and arabinose. Additionally, galactose can be substituted with an α‐l‐linked fucosyl group, forming fucogalactoxyloglucan. Xyloglucans exhibit significant structural and chemical diversity depending on the plant species, tissue type, and stage of maturation (Biel‐Nielsen et al., 2022).

The first scientist to describe pectin was Smolenski (1923), who identified it as a polymeric chain of galacturonic acid (GalA). Later, Kertesz (1951) defined pectin as a heterogeneous polysaccharide consisting of GalA esterified by methyl groups and linked cross to some neutral sugars. Around 1980, de Vries and his research group demonstrated that the neutral sugars are arranged in side chains within “hairy regions,” where about 90% of the GalA residues are composed only of the chain monomer (De Vries et al., 1983, 1982, 1981).

Actually, pectin is defined as a complex macromolecule composed of interconnected domains, the structure of which varies depending on botanical species, organs, cell types, stages of cell development, and specific locations within the cell wall. Pectin is categorized into two main families: galacturonans and rhamnogalacturonans.

Galacturonans are polymers primarily composed of α‐(1,4)‐linked at GalA units, which can be either branched or unbranched. Rhamnogalacturonans, on the other hand, have a backbone consisting of a diglycosyl repeating unit [2‐α‐l‐Rha‐(1,4)‐α‐d‐GalA‐(1)], where rhamnose residues are branched at the *O*‐4 (mainly) and *O*‐3 (sparsely) positions. These branches include monosaccharide chains containing arabinose and galactose residues in several blends (Ropartz & Ralet, 2020).

Pectins, cellulose, and xyloglucans are industrially obtained from apple pomace and citrus peels (Fu et al., 2006) but extracted under different conditions. For example, the pectins are extracted chemically with strong acids, such as hydrochloric, nitric, and sulfuric acids (Min et al., 2011), with a determinate pH value (1.4–3), temperature (60–100°C), and time (20–360 min) (Minjares‐Fuentes et al., 2014) and the world market request for pectin is more than 30,000 t annually and is growing of 4%–5% per year (Yeoh et al., 2008). In the food industry, pectin has many functional roles. For example, pectins with a high degree of methylation are involved in jams and jelly production, whereas low degrees of methylation are used for the preparation of juices and foods with a reduction of energy. It also represents use as an emulsifier and stabilizer and for the production of active packaging (Gavahian et al., 2021).

Xyloglucan, on the other hand, is extracted under very alkaline conditions (KOH or NaOH concentrated 1 or 4 M) as it binds tightly to the cellulose microfibrils through hydrogen bonds, cross‐linking them into a cellulose–xyloglucan network (Fu et al., 2006).

Generally, cellulose is extracted from wood through the Kraft process, where cellulose is separated from lignin and hemicellulose using sodium hydroxide and sodium sulfide. Another method for extracting cellulose from wood is the sulfite pulper process, which uses sulfurous acid and bisulfite ions to help rid the system of lignin (Garnett et al., 2024).

Thus, new trends involve the recovery of fiber from food waste to develop new foods with health benefits. Furthermore, the implementation of green technologies to limit or reduce the use of solvents that are toxic to humans and the environment represents a great opportunity to find the perfect combination of human and environmental well‐being.

## 4 FUNCTIONAL FOOD

Functional foods were initially defined by Roberfroid as foods resembling conventional food in appearance, intended to be consumed as part of the normal diet, but modified to provide physiological benefits beyond basic nutrition (Gobbetti et al., 2010). However, there is no universal definition of functional food (Roberfroid, 2002). Generally, functional foods are understood as foods that aim to promote human health due to the presence of bioactive compounds (Munekata et al., 2021). There is a growing concern over the consumption of junk food, a significant contributor to obesity. Additionally, the prevalence of diabetes has risen dramatically, from 108 million in 1980 to 422 million in 2014 (Taverna, 2018). Customers now consider various nutritional properties before making food purchases, such as the content of vitamins like ascorbic acid, tocopherol, vitamin of group B, minerals, and prebiotic and probiotic properties (Quan et al., 2020). Foods fortified with nutrients like omega‐3, phytosterols, and fiber are also sought after (Siró et al., 2008). In this scenario, therefore, formulating functional foods that provide health benefits can help reduce the risk of chronic diseases resulting from high consumption of saturated fatty acids and simple sugars. In addition, the possibility of adding food waste, which is still rich in bioactive compounds with beneficial properties, could help to reduce the amount of waste as a globally desirable environmental goal and revalorize waste.

**FIGURE 1:**
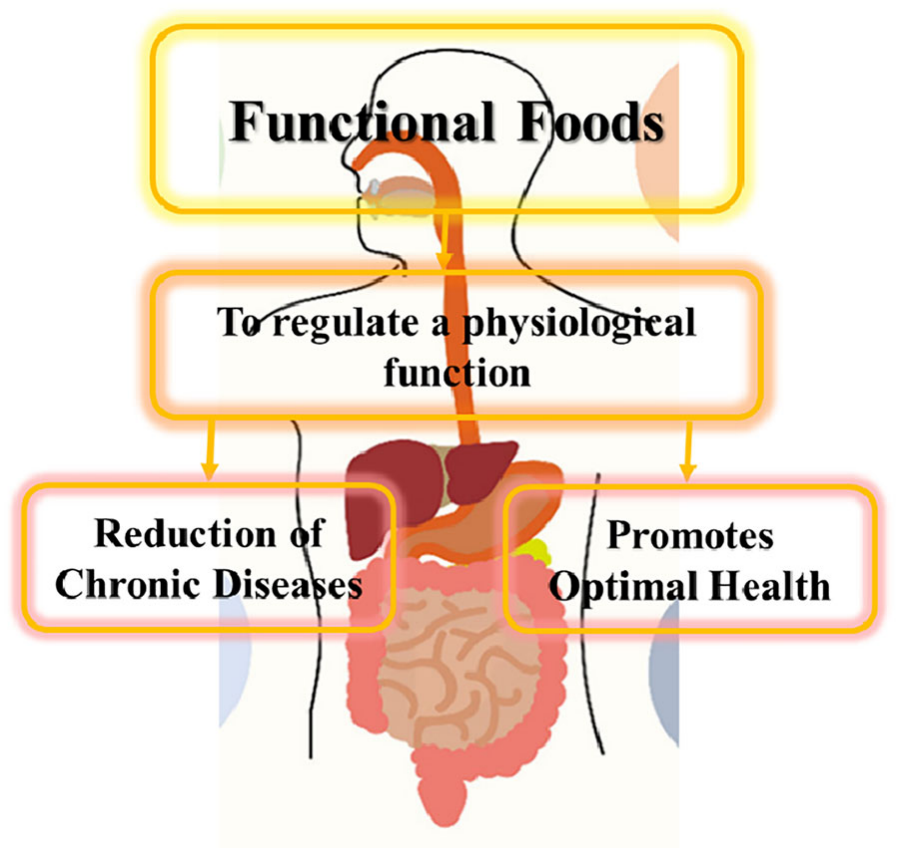
Aim of the functional foods.

Probiotics and prebiotics play a role in enhancing the gut microbiota and intestinal immune system, whereas polyphenols are valued for their anti‐inflammatory properties (Wan et al., 2019). Probiotic microorganisms offer various health benefits, including immune system stimulation (Cremonini et al., 2002), production of beneficial short‐chain fatty acids for conditions like colitis and cancer prevention (Cukrowska et al., 2009), and reduction of serum cholesterol levels and blood pressure (Rašić, 2003). Several studies have reported the potential use of probiotics in preventing and treating gastrointestinal (GI), urinogenital tract, and respiratory diseases (Gardiner et al., 2002). Studies indicate that oral doses exceeding 10^9^ colony‐forming units per day are necessary for maintaining bacterial balance (Wang et al., 2022), driving the increasing demand for probiotic functional foods due to heightened consumer awareness. Incorporating bioactive compounds like polyphenols into functional foods is often limited by challenges, such as low solubility, reduced stability, and limited bioavailability. To address these issues, the use of delivery systems, such as microemulsions, nano‐emulsions, and liposomes containing bioactive compounds, can offer effective solutions (Granato et al., 2020). Figure 1 reports the summarized function of functional foods.

## 5 GREEN TECHNOLOGIES: STATE OF THE ART

Green technologies do not have a clear definition, and as reported in the WIPO Green Strategic Plan 2019–2023 (WIPO, 2019), there is currently no global criterion for the classification of green technologies. Conceptually, they can be defined as a dynamic set of technologies that aims to achieve a technological system capable of reducing pollution, improving efficiency, protecting ecology, and promoting the harmonious coexistence between man and nature (Guo et al., 2020). Underlying green technologies is green chemistry, which involves the invention, design, and application of chemical substances and processes that aim to minimize or eliminate the use and production of toxic substances (Chemat et al., 2012). From a bio‐pharmacy perspective, developing new, environmentally friendly methods for extracting bioactive compounds from natural sources is essential. The six principles of green extractions described not as rules but as innovative examples to follow (Chemat et al., 2012) are:

1. Innovation through the use of renewable plant resources;
2. Use of alternative, biodegradable, non‐toxic, and non‐flammable solvents;
3. Reuse of energy released in extraction processes to reduce consumption and introduce possible innovative processes;
4. Production of co‐products (or byproducts) and not waste;
5. Reduce the number of operations to implement safe and controlled processes, reducing costs and increasing energy efficiency;
6. The extracted product should be unaltered, biodegradable, and uncontaminated.

The main extraction technologies based on green chemistry involve the use of ultrasound (US), microwave, supercritical fluids (SFs), enzymes, natural deep eutectic solvents (NaDESs), and pressurized fluids (Abdelrahman et al., 2023; Al Khawli et al., 2019; AlYammahi et al., 2023). These methods are described in the paragraphs below, and Table 1 shows the quantities of food waste produced from raw materials and studies using individual green technologies or combinations thereof for food waste recovery.

**TABLE 1:** Waste produced from raw material and application of individual green technologies to recover bioactive compounds.

Food waste: tomato Waste from raw material: 2%–6% composed of 44% seeds and 56% skin and pulp (Romano et al., 2020)		
Type and extraction conditions	Main results	References
UAE (pH 1.5, SLR 1:30, 80°C, 20 min) vs. MAE (SLR of 1:20, pH = 1.5, 300 W, 10 min)	MAE ↑ lycopene content	Lasunon and Sengkhamparn (2022)
UAE (90 W, SLR 1:35, 30 min)	↑ Lycopene yield	Kumcuoglu et al. (2014)
MAE (80°C, EtOH 63%, 15 min) vs. UAE (5 min, EtOH 61%, 85% amplitude)	MAE ↑ TPC, DPPH, ABTS, FRAP chlorogenic acid, rutin, and naringenin content	Solaberrieta et al. (2022)
EAE (40°C, 5 h, enzyme:substrate 0.2 mL/g)	↑ Lycopene	Catalkaya and Kahveci (2019)

Food waste: pomegranate peels Waste from raw material: 50% of the whole fruit (El Barnossi et al.,^2021^)		
Type and extraction conditions	Main results	References
MAE (50% EtOH, 4 min, 600 W, SLR 1:60 g/mL) vs. UAE (H_2_O, 10 min, amplitude 52 W, SLR 1:32.2 g/mL)	MAE ↑ TPC and punicalagin content	Kaderides et al. (2019)
EAE (cocktail of enzyme, 49°C, 85 min, pH 6.7) + SFE (55°C, 300 bar)	↑ Antioxidant activity and phenolic acids	Mushtaq et al. (2015)
NaDES (ChCl:LA, 35% LA in NaDES mix, 20 w/w NaDES/peels, 25 min, 45°C)	TPC ↑ 54.6% compared to non‐optimized conditions and ↑ 84.2% compared to EtOH	Bertolo et al. (2021)

Food waste: spent of coffee Waste from raw material: 1 t of raw green coffee produces almost 650 kg of spent coffee grounds (Stylianou et al., 2018)		
Type and extraction conditions	Main results	References
NaDES (ChCl:Prop vs. Bet:Teg)	ChCl: Prop ↑ polyphenols and caffeine content Bet: Teg ↑ AA	García‐Roldán et al. (2023)
MAE (68.9% EtOH, SLR 1:16.7, 90 W and 4.5 min)	Yield: 6.98%, TPC: 117.7 mg GAE/g and AA 143.8 µmol TE/g	Coelho et al. (2021)
Supercritical CO_2_ (265 bar, 55°C, moisture content of 55 wt%)	Lipid recovery: 2.67 wt%; Caffein recovery: 5.36 wt%	Vandeponseele et al. (2022)

Food waste: lemon waste Waste from raw material: peels, membranes, and seeds make up almost 50% of the fruit (Gómez‐Mejía et al., 2019)		
**Type and extraction conditions**	**Main results**	**References**
UAE (EtOH 63.93%, SLR 1:40 g/mL, 15.05 min, 77.79% of amplitude) vs. MAE (EtOH 48% EtOH, SLR 1:28 g/mL, 123 s, 400 W)	MAE ↑ AA with a shorter working time and lower solvent consumption	Dahmoune et al. (2013)
UAE (60°C, 45 min, acidified extraction with HNO_3_) vs. MAE (360 W, 1 min acidified extraction with HNO_3_)	Pectin yield: 9.61% for UAE/9.71% for MAE	Karbuz and Tugrul (2021)

Food waste: orange waste Waste from raw material: 50% of the weight of the fruit (Rezzadori et al., 2012)		
Type and extraction conditions	Main results	References
Supercritical CO_2_ (30 MPa, 40°C) + UAE (68°C, 45 min, SLR 1:41 g/mL, EtOH: H_2_O 61%)	The optimal SFE resulted in a yield of 3.23%, the optimal UAE resulted in 23.0 µg/mL hesperidin.	Jokić et al. (2020)
EAE (cellulase enzyme, 2% (w/v) substrate, 1% (v/v) enzyme, pH at 4.8) + UAE 60 W and a duty cycle of 70%, 6 h, 50°C)	Maximum amount of fermentable sugars: 30 g/L	Utekar et al. (2021)

Food waste: chestnuts shells Waste from raw material: 10% of the total chestnut mass (Husanu et al., 2020)		
Type and extraction conditions	Main results	References
UAE (70°C, 40 min, 50% of amplitude, SLR 0.05 g/mL of water)	Extraction yield: 16%; TPC: 393.1 mg GAE/g dw; DPPH, IC50: 44.1 µg/mL; FRAP, IC50: 32.0 µg/mL; ABTS, IC50: 65.4 µg/mL; ellagic acid: 40.4 µg/mg, caffeic acid derivative: 15.4 µg/mg, epigallocatechin: 15.3 µg/mg	Lameirão et al. (2020)
NaDES (ChCl:LA containing 20% w/w water, 120°C, 24 h)	Lignin extraction yield: 19%; ellagic acid: 0.9% w/w; TPC: 0.35 mg GAE/mg of sample; DPPH, EC50: 0.05 mg/mL; FRAP: 0.08 mg TE/mg of sample	Argenziano et al. (2022)

*Note*: ↑: increase; Prop: 1,2‐propanediol; Bet:Teg: betaine:triethylene glycol.

Abbreviations: AA, antioxidant activity; ChCl, choline chloride; EAE, enzyme‐assisted extraction; LA, lactic acid; MAE, microwave‐assisted extraction; NaDES, natural deep eutectic solvent; SLR, solid:liquid ratio; UAE, ultrasound‐assisted extraction.

### 5.1 Microwave‐assisted extraction

Microwaves are electromagnetic waves with frequencies from 300 MHz to 300 GHz capable of activating the rotational energy levels of molecules. These electromagnetic radiations generate an electric field by starting an ionic conduction, based on the transfer of ions and electrons with an electrophoretic process. At these frequencies, there is the production of a rapid movement of polar compounds and charged ions (Al‐Ghouti et al., 2021). On an industrial level, microwaves are used for drying because they have lower energy consumption and products with better sensory attributes for cooking, which succeeds in preserving nutritional quality while significantly reducing anti‐nutritional factors, and also to sterilize food from bacteria, microwaves can effectively use to ensure the microbiological safety of food products. There are no noticeable changes in antioxidant activity, color, and bioactive components due to the disruption of enzyme activity and the short exposure time (Guo et al., 2017).

**FIGURE 2:**
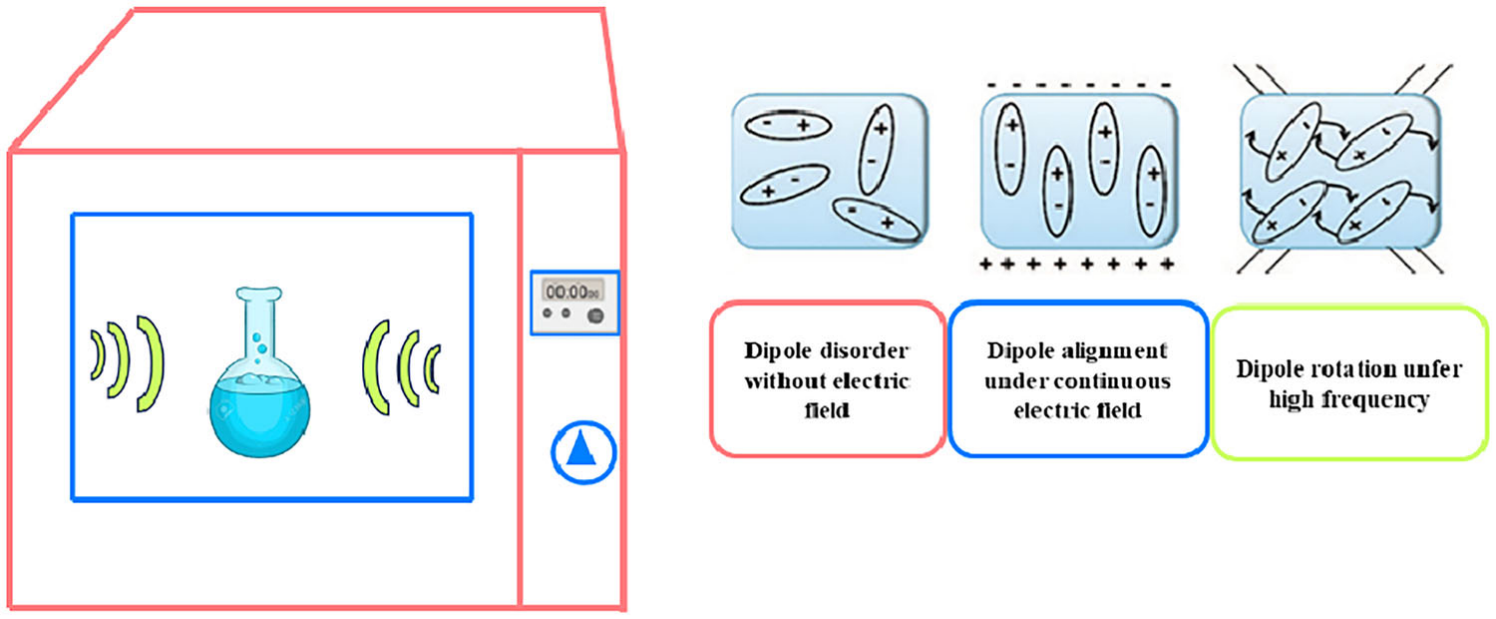
Representation of microwave‐assisted extraction (MAE).

A schematic representation of microwave operation in Figure 2 was reported.

Microwaves can penetrate the food matrices to interact with polar compounds like polyphenols, causing a pressure increase inside cells, determining the break of cell walls and release of phytocompounds. So, consequently, extraction with microwaves determines a reduction of time and energy compared to conventional heating methods (Ekezie et al., 2017). Anyway, the use of high‐power microwaves causes an increase in extract yield but could reduce the polyphenol content due to their degradation.

The moisture content in the matrix can influence the ability of the matrix to absorb microwaves and facilitate heating. Furthermore, the water can also cause swelling of the matrix and/or affect analyte–matrix interactions, thus making extraction more difficult (Vinatoru et al., 2017). Furthermore, the presence of bonds between molecules of interest such as polyphenols and water is a factor to be taken into account for microwave‐assisted extractions (Liu et al., 2020). In this way, Lasunon et al. (2021) have evaluated the recovery of hydrophilic and lipophilic bioactive compounds from tomato waste using microwave‐assisted extraction (MAE), showing that to extract the hydrophobic component, the optimal condition was 300 W for 60 s, obtaining thigh trans‐lycopene and beta‐carotene content (5.74 mg lycopene/100 g and 4.83 mg beta‐carotene/100 g), whereas high DPPH radical scavenging value was reported at 180 W for 90 s. Regarding the hydrophilic components, the optimal condition with the highest total phenolic content (280.10 mg GAE/100 g) and total flavonoid content (9832.52 mg CE/100 g DM) was reported at 180 W for 90 s and at 450 W for 30 s, respectively. Furthermore, Petrotos et al. (2021) evaluated the best conditions of MAE to extract polyphenols from the pomegranate pomace (PP), a waste of the pomegranate juice industry, showing that the optimal extraction condition for the recovery of total polyphenol (TP) content (209,703 mg GAE/kg of raw pomegranate) juice industry was found at 4961.07 W, with a water/PP ratio of 29.90 and an extraction time of 119.53 min.

Prakash Maran et al. (2013) evaluated the MAE of pectin from citrus peel optimizing the following parameters: power (160–320–480 W), irradiation time (60–120–180 s), pH (1–1.5–2), and solid–liquid ratio (1:10–1:20–1:30 g/mL). The optimal extraction conditions and the highest yield of pectin (19.24%) were obtained at 422 W, 169 s, pH of 1.4, and a solid–liquid ratio of 1:16.9 g/mL. The pectin yield was increased with time, but at more than 125 s, there was a decrease, and this could be explained by the excessive time exposure so the microwave can cause the degradation of pectin.

### 5.2 Supercritical fluid extraction

SF is a substance that exists at a temperature and pressure above its critical point (Ahangari et al., 2021). SF extraction (SFE) is an innovative technology known for its environmental friendliness (Uwineza & Waśkiewicz, 2020) and is very employed for extracting beneficial components from vegetable matrices (Akgün et al., 2014). Among SFs, carbon dioxide (CO_2_) is most used for its low critical point (74 bar and 32°C) because it is considered non‐toxicity, non‐flammability, available in high purity at relativity low cost, and easy to remove from the extracts (Araújo et al., 2019). CO_2_ is an apolar lipophilic compound, and the solubility of bioactive compounds with high polarity is weak in this fluid. So, the addition of a polar co‐solvent, such as ethanol, is necessary to increase the solubility of polar bioactive compounds (Muangrat & Pongsirikul, 2019). At the industrial level, CO_2_ has several applications, such as the extraction of targeted bioactive compounds from various food matrices, microencapsulation or extrusion to produce fine particles, inactivation of pathogenic and spoilage microorganisms, and endogenous enzymes for food preservation, but one of the main applications is the decaffeination of coffee (Wang et al., 2021) and for the extraction of chlorophyll and carotenoids (Morcelli et al., 2021).

**FIGURE 3:**
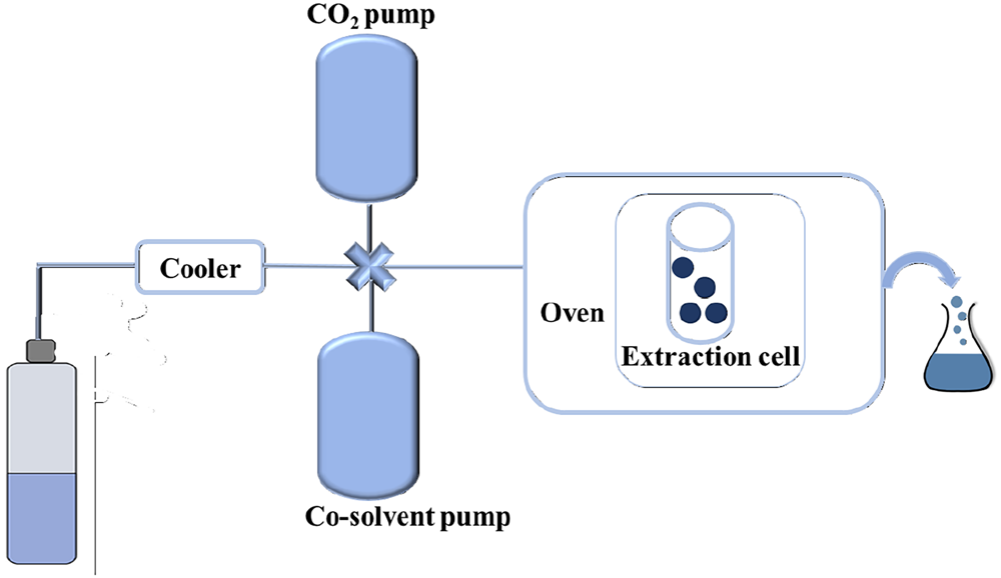
Schematic supercritical fluid extractor (SFE).

The principle of extractor operation in Figure 3 was shown.

Different studies reported the use of SFE for bioactive compound extraction. For instance, the recovery of lycopene and carotene from tomato waste (peels and seeds) using supercritical CO_2_ has been extensively investigated (Romano et al., 2020; Squillace et al., 2020). Urbonavičienė et al. (2018) using supercritical CO_2_ recovered from tomato byproducts six isomers of lycopene (Isomers 15; 13; 9; 7 cis and 1; 5 trans isomers) and evaluated with an in vitro assay the antioxidant capacity of obtained extracts on murine macrophage J774 cell culture. Their findings demonstrated that the extract reduced the production of H_2_O_2_ in the murine macrophage J774 culture by direct scavenging.

Argun et al. (2022) recovered the orange processing wastewater with the use of supercritical CO_2_, reporting that the TP content increased with increasing extraction pressure, indicating a correlation between pressure and extractive power (Romano et al., 2023).

### 5.3 Ultrasound‐assisted extraction

US, defined as mechanical waves that propagate in an elastic medium such as liquids, operates within a frequency range of 20 kHz–10 MHz. The key phenomenon associated with US is cavitation, involving the formation, growth, and implosion of bubbles during wave propagation through the medium. Near solid surfaces, collapsing cavitation bubbles generate micro‐jets and shock waves directed toward the surface, leading to disruption of cell walls (Marić et al., 2018).

In the food industry, US has several applications as an alternative to conventional thermal pasteurization of food products because it allows microbial inactivation to be achieved and the nutritional and organoleptic characteristics (texture, color, taste) of the food to be maintained, with the advantage of reducing processing times. In addition, they have also been used as adjuvants to improve operations, such as filtration, cooking, degassing, cutting, drying, meat tenderization, homogenization, and crystallization (Welti‐Chanes et al., 2017).

**FIGURE 4:**
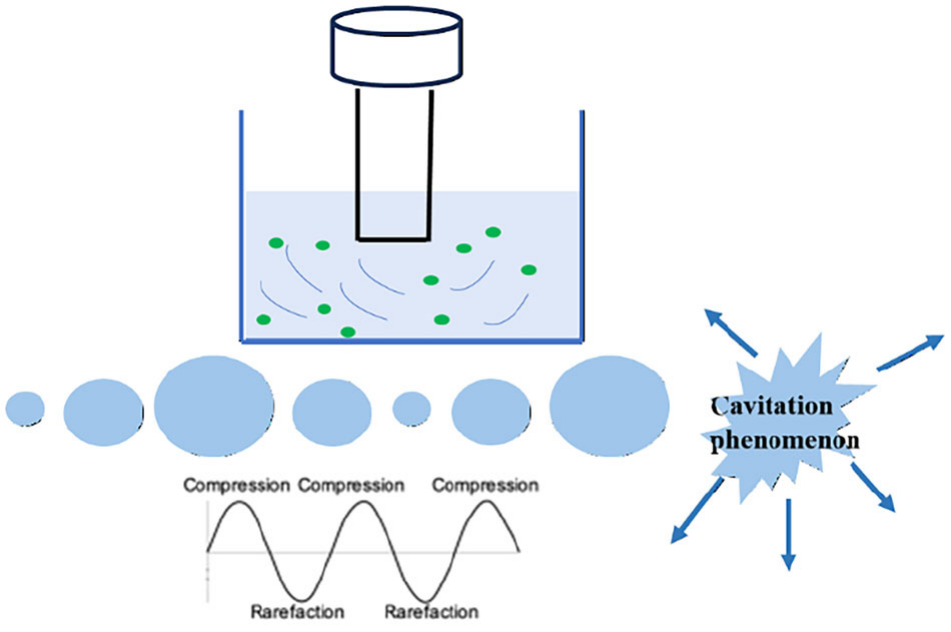
Principle of ultrasound‐assisted extraction (UAE).

US‐assisted extraction (UAE), whose principle of operation is reported in Figure 4, is a technique used to recover bioactive compounds from food waste. Garcia‐Castello et al. (2015) utilized UAE to extract flavonoids from grapefruit peels, investigating the impact of variables, such as % EtOH, temperature, and time on phenolic compound yield and antioxidant activity. Optimal conditions identified were 40% ethanol, 25°C, and 55 min, balancing economic and environmental considerations effectively.

UAE was used by Grassino et al. (2016) to evaluate the recovery of pectins from tomato waste. They reported that UAE is a valid alternative to recover pectins compared to conventional extraction for its small time of extraction (from 15′ to 90′). In fact, the percentage of pectins recovered from tomato waste by conventional extraction methods was relatively high, 21.1% and 15.1%, at 60 and 80°C, respectively. In comparison, the pectins extracted by UAE ranged from 15.2% to 17.2% and 16.3% to 18.5% at 60 and 80°C, respectively.

Minjares‐Fuentes et al. (2014) evaluated UAE of pectin from grape pomace using citric acid as the key factor for extraction, assessing key parameters such as molecular weight (MW) and degree of esterification (DE), which are important for gelling and thickening. The results indicated that the yield of extracted pectin ranged from 3.2% to 29.4%, with the maximum yield achieved at 75°C, 40 min, and pH 2.0; the molecular weight (MW) of the pectin extract ranged from 111 to 205 kDa; pectins extracted after 20 min at 55°C and pH 2.0 exhibited the highest molecular weight; the DE ranged from 20.1% to 61.2%, with the highest DE value observed at 75°C for 40 min and pH 1.0. In addition, the yield obtained under optimal UAE conditions was 20% higher compared to extraction without US at the same temperature, time, and pH. In conclusion, pectins extracted via UAE demonstrated a higher molecular weight compared to conventional methods. These results underscore the effectiveness of UAE in optimizing pectin extraction yield, molecular weight, and degree of esterification, highlighting its superiority over conventional extraction methods under certain conditions.

Fu et al. (2006) compared the UAE of xyloglucan from apple pomace with the conventional method using concentrated alkali (NaOH or KOH 1 or 4 M). Their study showed a threefold decrease in time as a result and estimated a liquid:solid ratio of 34.4:1 (v/w), KOH 3.3 M, 160 W, and a time of 2.5 h. The authors showed that xyloglucan extracted from apple pomace was increased with the increase of UAE time and reached the highest point at 4 h, but if the extraction was longer than 4 h, there was a decrease in extraction.

### 5.4 Enzyme‐assisted extraction

In the food and industrial sectors, the application of enzymes is essential for some production processes and finds countless applications, such as the production of glucose syrups, high fructose corn syrups, maltose syrups, lactose‐free milk or cheeses, clarification of fruit juices, for food prevention, and cheese production (Raveendran et al., 2018).

The use of enzymes makes it possible to improve the extraction process by hydrolyzing the cell wall of the matrix. As a result, disruption of the wall allows increased cell permeability (Marić et al., 2018). Hydrolytic enzymes such as cellulase and pectinase are widely used to degrade the cell wall and consequently increase the release of intracellular compounds.

Kaur et al. (2023) used UAE combined with enzymatic extraction (US‐assisted enzymatic extraction [UAEE]) to recover fiber from kinnow peel because both extractions can be considered a simple and green technique. The enzymatic digestion was performed by α‐amylase, amyloglucosidase, and protease, and the result showed that the total dietary fiber was 52.04 for UAE and 60.9 UAEE using an amplitude of 38%, 44°C, 13 min, and a liquid‐to‐solid ratio of 40 mL/g. Lombardelli et al. (2021) have recovered the betalains from unsold red beets with an enzymatic mix composed of 37% cellulase, 35% xylanase, and 28% pectinase. The optimal extraction conditions were 25 U/g total dose of enzymatic mix, 25°C as extraction temperature, and 240 min as extraction time. Recently, Vilas‐Franquesa et al. (2024) evaluated the potential application of different enzymatic blends and probiotic strains (*Lactiplantibacillus plantarum* and *Bifidobacterium animalis*) to increase the solubilization of polyphenols in aqueous extracts from mango peels. The authors hypothesized that the enzymatic pretreatment with β‐glucanase, pectinase, endoglucanase, and a mix of them can enhance the bacteria's growth and the fermentation of mango peels, increasing the recovery of phenolic compounds like gallic acid and mangiferin compared to non‐enzymatically treated and uninoculated samples. The use of probiotic strains and β‐glucanase enzyme treatment increased gallic acid content by about 150% compared with not inoculated samples. The use of pectinase and probiotic bacteria causes an increase of 1500% concerning the not inoculated samples.

Finally, Kaur et al. (2023) extracted fiber from kinnow peels by UAE and UAEE. The dietary fiber obtained after ultrasonic treatment ranged from 38.88% to 51.78%, but with the addition of enzymatic treatment, there is an increase of 12.45%–24.47% in the dietary fiber recovery.

Biel‐Nielsen et al. (2022) explored the use of depectinized industrial citrus (lemon and orange) to produce human milk oligosaccharides through enzymatic transfucosylation. They converted fucosylated xyloglucan from depectinized residues into 2′‐fucosyllactose, an oligosaccharide found in human milk. Approximately 35%–36% and 48%–51% of the initial fucose content in depectinized lemon and orange residues, respectively, were recovered as fucosylated xyloglucan using enzymatic or treatment with alkali. Transfucosylation was conducted using fungal fucosidase, resulting in yields of 10.2%–11.4% for enzymatically treated samples and 6.5%–7.4% for alkali‐treated samples (for orange and lemon, respectively).

In another study, Kaur et al. (2023) extracted fiber from kinnow peels using UAE and UAEE. The dietary fiber recovery after ultrasonic treatment ranged from 38.88% to 51.78%. With the addition of enzymatic treatment, there was a notable increase of 12.45%–24.47% in the recovery of dietary fiber. This approach demonstrates the effectiveness of combining US with enzymatic treatment to enhance dietary fiber extraction from kinnow peels.

**FIGURE 5:**
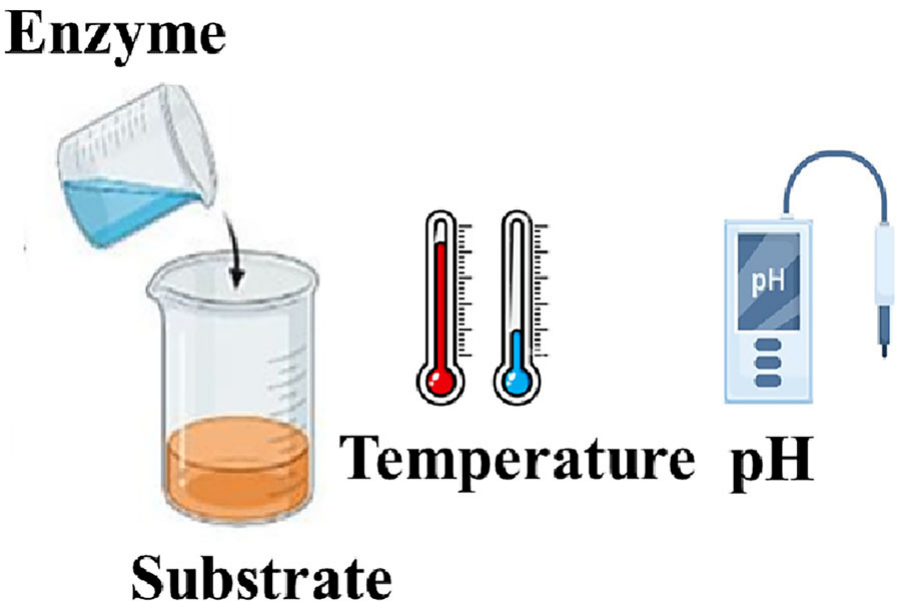
Schematic representation enzyme‐assisted extraction (EAE) and factors that influence it.

In Figure 5, a representative factor that can influence enzyme‐assisted extraction is reported.

### 5.5 Natural deep eutectic solvents

**FIGURE 6:**
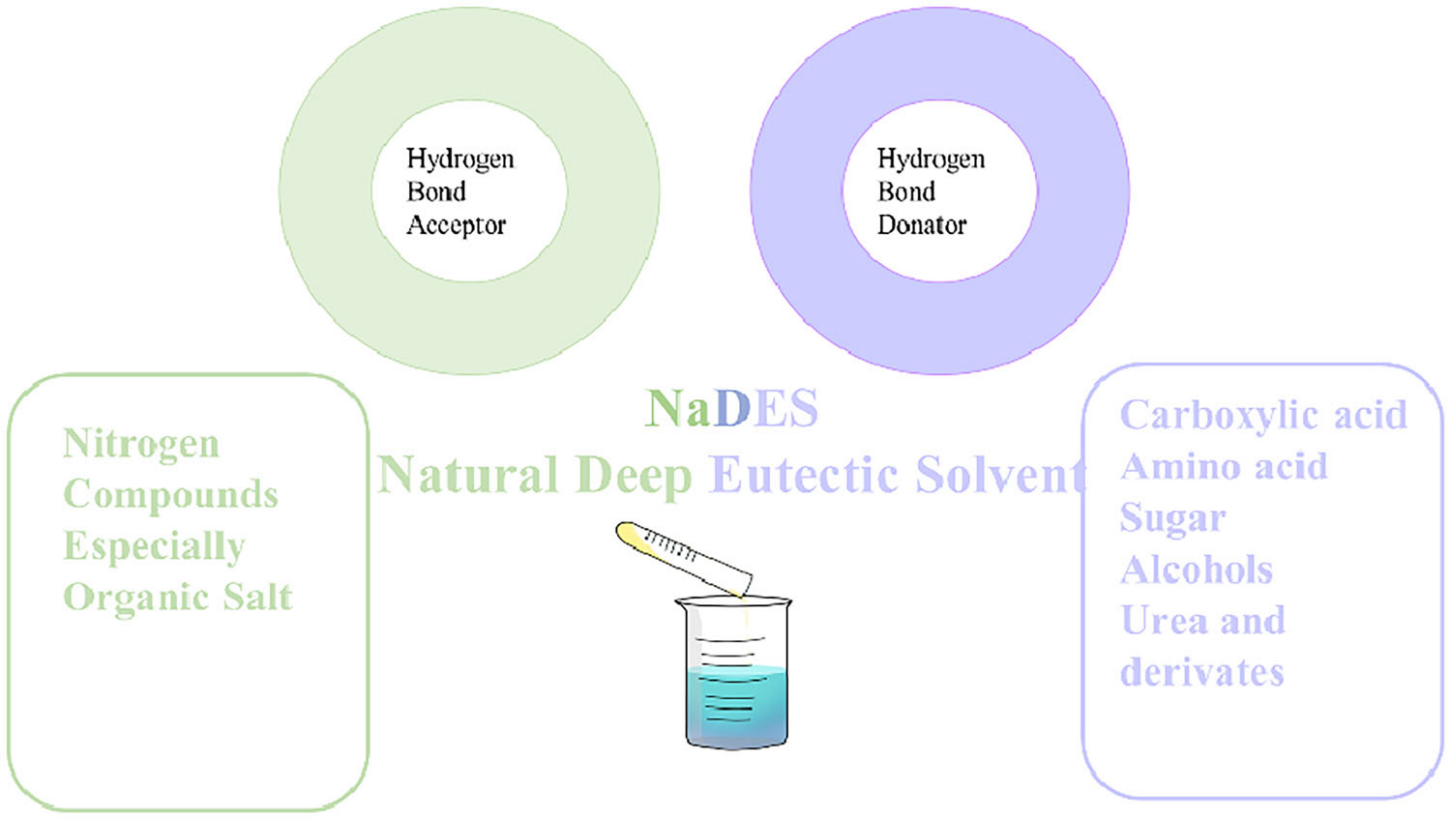
Main chemical classes for the formation of natural deep eutectic solvent (NaDES).

DESs are defined as liquid eutectic mixtures composed of at least two components: a hydrogen bond donor (HBD) and a hydrogen bond acceptor (HBA). These components can form intermolecular hydrogen bonds and van der Waals interactions. When the constituents of DES are natural, they are referred to as NaDESs (Tzani et al., 2021). DES remains in a liquid state at low temperatures, is miscible with water, nonflammable, and highly viscous (El Kantar et al., 2019). Typically, the HBA in DES is represented by quaternary ammonium salts such as choline chloride (ChCl), paired with various HBDs including amines, alcohols, carboxylic acids, sugars, and vitamins (Rukavina et al., 2021). NaDES has been extensively studied for its efficacy in extracting bioactive compounds, such as polyphenols, terpenoids, and alkaloids (Lanjekar & Rathod, 2021). The key advantages of NaDES include low volatility, negligible toxicity, miscibility with water, adjustable viscosity, the ability to form solutions with a broad range of polarities, and the potential to recover the solvent from the final product (Bertolo et al., 2021). Several studies have utilized NaDES, as detailed in Table 2. Furthermore, Figure 6 provides a schematic representation of a NaDES mixture, illustrating its composition and structure.

**TABLE 2:** Types of natural deep eutectic solvent (NaDES) used to recover food waste.

NaDES	Molar ratio	pH	Viscosity (mPa s)	Utilization	References
Glucose:choline chloride Fructose:choline chloride Xylose:choline chloride Glycerol:choline chloride Malic acid:choline chloride	1:2 1:1.9 1:2 2:1 1:1	NR	NR	Recovery of polyphenols from grape skin	Radošević et al. (2016)
Glucose:lactic acid:H_2_O Glucose:lactic acid:H_2_O Glucose:lactic acid Glycine:lactic acid:H_2_O Glycine:lactic acid	1:6:6 1:5:3 1:5 1:3:1 1:9	0.56 0.48 0.31 2.52 1.85	68.99 181.1 416.90 112.50 166.50	Recovery of pectins from pomelo peels	Liew et al. (2018)
Ascorbic acid:choline chloride	1:1.2 1:2 1:2.5	NR	∼18.00 12.54 ∼10.00	Recovery of antioxidant molecules	W. Liu et al. (2018)
Glucose:lactic acid Glucose:citric acid Citric acid:fructose	1:5 1:1 1:1	NR	NR	Phenolic compound recovery from the olive cake, pear waste, onion, and tomato waste	Fernández et al. (2018)
Levulinic acid:methyl urea:choline chloride	1:1:1	NR	NR	Recovery of polyphenols from citrus waste	Xu et al. (2019)
Glucose:choline chloride Sucrose:choline chloride Glycerol:choline chloride Lactic acid:choline chloride Citric acid:choline chloride	1:1 1:1 1:1 1:1 1:1	4.78 5.53 4.45 1.44 <1	466.60 392.26 31.70 26.30 295.50	Recovery of phenolic compounds from pomegranate peels	Bertolo et al. (2021)
Malic acid:choline chloride Urea:choline chloride Fructose:choline chloride	1:1 1:1 1:1	2.00 5.90 5.32	57.56 20.95 69.69	Recovery of polyphenols from sour cherry pomace	Popovic et al. (2022)
Glycerol:choline chloride Glucose:choline chloride Citric acid:choline chloride Proline:choline chloride	2:1 1:1 1:1 1:1	NR	NR	Recovery of polyphenols from coffee husk	Maimulyanti et al. (2023)
Choline chloride:glucose Choline chloride:glycerol Choline chloride:ethylene glycol Choline chloride:fructose:sucrose Glucose:ethylene glycol Sorbitol:ethylene glycol Glucose:glycerol	1:1 1:2 1:2 1:1:1 1:2 1:2 1:2	Reported in function of water content (30%, 50%, or 80%)	NR	Utilization of orange peels to synthesize (R)‐phenylethanol and recover d‐limonene and polyphenols	Panić et al. (2021)
Sodium acetate:lactic acid Sodium acetate:lactic acid Sodium acetate:glycerol Sodium acetate:glycerol Glycerol:Na‐K tartrate:H_2_O Glycerol:Na‐K‐tartrate:H_2_O Glycerol:choline chloride Glycerol:choline chloride Glycerol:choline chloride Glycerol:choline chloride Ethylene glycol:choline chloride Ethylene glycol:choline chloride Ethylene glycol:choline chloride	1:3 1:5 1:3 1:5 5:1:3 5:1:4 1:1 2:1 3:1 4:1 2:1 3:1 4:1	NR	NR	Recovery of fiber and polyphenols from chestnut wood fiber	Moccia et al. (2022)
Lactic acid:choline chloride Lactic acid:choline chloride Tartaric acid:choline chloride Tartaric acid:choline chloride Glycolic acid:choline chloride Oxalic acid dihydrate:choline chloride Urea:choline chloride Malic acid:choline chloride Maleic acid:choline chloride Malonic acid:choline chloride Glucose:lactic acid Fructose:lactic acid	2:1 9:1 1:1 1:2 3:1 1:1 2:1 1:1.5 1:1 1:1 1:5 1:5				

*Note*: ∼: approximately.

Abbreviation: NR, not reported.

## 6 FUNCTIONAL FOOD FROM FOOD WASTE

### 6.1 Bakery products with food waste extract

Bakery products are widely valued and globally consumed. Baked goods constitute a fundamental element of the diet due to their regular consumption in significant amounts. Indeed, they are linked to all moments of the day, ranging from breakfast and snacks to main meals (Nicolosi et al., 2023). Today's consumers are constantly looking for innovative products and are increasingly attentive to both the nutritional profile and sustainability of food (Mitelut et al., 2021). So, the formulation of functional baked goods could be a crucial strategy to decrease the likelihood of developing chronic diseases in addition to basic nutritional functions (Martins et al., 2017; Qian et al., 2021). As reported by Lin (2022), bakery products are currently regarded as convenient dietary vehicles for the introduction of nutritional ingredients into people's diets, including, for example, fibers and numerous other bioactive compounds. Among extracts obtained from food waste, those obtained from coffee waste have been widely used. In fact, Dauber et al. (2024) formulated cookies using coffee silverskin extract obtained by UAE at 180 W and 60 min. The addition of 0.8% and 1.2% of the coffee silverskin extract to the cookie formulation resulted in an increase of 13.8% and 31.2% for total phenolic compounds (TPCs) and an increase of 21% and 45.5% of Trolox equivalent antioxidant capacity. Azuan et al. (2020) formulated cookies with various percentages (0.27%–0.57%–0.80%–1.07%–1.33%) of spent coffee grounds extract by UAE (50 kHz, 400 W, 40°C, 1 h), showing that the increment of extract added in cookies leads to an increase in fiber content compared to control. In addition, the inclusion of spent coffee ground extract in the cookie preparation exhibited an increase in TPC and antioxidant power measured by DPPH and ferric ion reducing antioxidant power (FRAP) assays. The extracts of coffee are also used, such as butter replacer butter, in cookie production, as reported by Meerasri and Sothornvit (2022), who have formulated healthy cookies by replacing butter with oil extracted from coffee grounds in substitution of 10%, 20%, and 30%. The addition of spent coffee ground oil as a substitute for butter greatly affected the rheological properties of the cookies, leading to a decrease in hardness and thus causing the cookie to soften. At the sensory level, the substitution of up to 20% ensured the acceptability of the product. Furthermore, Imeneo et al. (2021) have formulated cookies fortified with lemon peels and with the extract of lemon peels obtained by UAE. The enrichment with lemon peels increased by 126% TPC and by 23% antioxidant activity compared to the control, whereas the fortified cookies with extract of lemon peels increased by 100% TPC and by 29% antioxidant activity compared to the control. Kaur et al. (2023) formulated biscuits enriched with 5%, 10%, and 15% w/w of the fiber extracted by UAEE to obtain high‐fiber cookies because, according to some studies, consumption of fiber‐rich foods can bring health benefits such as management of blood glucose and cholesterol levels. The incorporation of 5% dietary fiber into the cookies did not impact their taste, whereas adding 15% resulted in a slightly bitter taste and introduced some extraneous flavors. Despite this, the overall acceptability of all the cookies remained high, with ratings exceeding 7. These findings suggest that citrus dietary fiber can be effectively utilized to create high‐fiber cookies without compromising taste and acceptability. Báez et al. (2021) have used a cellulase‐ and protease‐assisted brewer's spent grain enzymatic extraction. The authors show that the optimal extract (TPC, ABTS, and ORAC assays) was obtained with 0.1% protease and 0% cellulase. The hydrolysate obtained was freeze‐dried and then used for the production of bread as a 20% replacement for wheat flour. The addition of the hydrolysate allows about 146% increase in fiber content and also an increase in TPC content (0.27 mg GAE/g for the control and 0.47 mg GAE/g for the enriched bread) and also an increase in antioxidant activity measured by ABTS assay of about 17%. Thus, it was possible to recover food waste by enzymatic hydrolysis and implement it in the formulation of an enriched bread.

### 6.2 Fermented beverage with food waste extract

Nowadays, fermented milk represents the most widely used matrix for the development of commercial functional beverages (Lamsal & Faubion, 2009). The study carried out by Al‐Hindi and Abd El Ghani (2020) involved the development of a fermented milk drink with added polyphenols extracted from pomegranate peel and probiotic strains *L. plantarum DMSZ 20079* and *Bifidobacterium longum* subsp. *longum DSMZ 200707*. In vitro and in vivo studies showed a significant reduction in cholesterol, LDL, and triacylglicerol levels in rats after 30 days. In addition, there was a decrease in TBARS levels and an increase in antioxidant enzyme activities (SOD, GST, GPx, CAT, and TAC) and GSH levels compared with the control group. Aliakbarian et al. (2015) used polyphenols from olive and grape pomace to produce milk fermented using culture of two different microorganisms, *Streptococcus thermophilus TA040* and the probiotic *Lactobacillus acidophilus* (*LAC4*), to obtain a probiotic beverage. Extracts from the fermented samples were tested for the total content of TPs, and their antiradical power (ARP) was evaluated. After 7 days of cold incubation, the TP content increased by 14.41% and 10.18% for fermented milk with the addition of grape pomace and olive pomace, respectively. Increasing storage up to 50 days led to a decrease in TP content for drinks with added extracts, whereas the TP content of control samples remained constant during storage. For the ARP, samples containing olive pomace extracts showed a value higher of 97.67% than the control.

Iriondo‐DeHond et al. (2020) have evaluated the use of extract from grape pomace (seeds and peels) for the production of yogurts incorporated with inulin and fructo‐oligosaccharides (FOS) to develop a product with antioxidant and anti‐diabetic properties. The addition of 5 mg/mL of extract increased antioxidant activity by ABTS assay of 212%, 274%, and 380% for yogurt additional with grape pomace extract, seed extract, and skin extract, respectively, compared to the control. The yogurt added with winery byproduct extracts and fiber showed similar physicochemical, textural, and microbiological properties during the shelf life. Moreover, consumer testing indicated that the enriched yogurts received the highest acceptance scores, suggesting that grape skin extract could be potentially utilized as a natural colorant in food products. Servili et al. (2011) used olive vegetation water to produce and characterize a fermented and functional drink enriched in polyphenols with γ‐aminobutyric acid (GABA) production from lactic acid bacteria indigenous to the human GI tract. The authors developed two milk‐based functional beverages enriched with phenolic extracts at the concentrations of 10 and 20 mg/100 mL from olive planting water and fermented with GABA, producing *Lactobacillus plantarum* C48 and *Lactobacillus paracasei* 15N. During fermentation, the total concentration of free amino acids and GABA increased (63–67 mg/L). In addition, there was an increase in 3,4‐dihydroxyphenolethanol (3,4‐DPEA) and a decrease in 3,4‐dihydroxyphenylethanol‐elenolic acid dialdehyde (3,4‐DHPEAEDA), *p*‐hydroxyphenylethanol (*p*‐HPEA), and verbascoside with fermentation probably due to hydrolysis of 3,4‐DHPEAEDA. The hydrolysis was favored because of the low pH or because of the esterase activities of lactic acid bacteria. The work done by Salazar‐Bermeo et al. (2023) involves the recovery of persimmon through UAE in combination with NaDES in order to have a fiber‐rich fraction and a eutectic fraction with tannins, polyphenols, and carotenoids. A NaDES mixture composed of citric acid:malic acid:water in a molar ratio of 1:1:10 was combined with US for 15 min and 40 Hz. The authors formulated three types of beverages (isotonic, energy, and dairy‐based) containing 3% fiber recovered from persimmon pulp. The authors show how fiber‐enriched fractions have greater antioxidant activity using ABTS and DPPH assays. Furthermore, following in vitro digestion, the fiber extracted from persimmon pulp showed that probiotic strains of *Lactobacillus casei* and *Lactococcus lactis* are able to ferment the treated fiber more easily than the untreated one. Finally, the lactic‐based drink formulated and added with fiber obtained from green extraction showed an acceptability of 50% higher than the control.

## 7 CONCLUSION AND FUTURE PERSPECTIVE

The recovery and reuse of waste is a problem of increasing interest for both consumers and companies. The combination of green technologies for the recovery of bioactive compounds represents a future trend to always pay greater attention to the environmental footprint. The use of green extracts obtained from food waste for the production of functional foods is not yet widely explored. The bioaccessibility of compounds is a concept related to the type of nutrient, the type of food matrix, the technological processes undergone by the matrix, and individual variability, whereas bioavailability depends on bioaccessibility and individual variability. The use of bioactive compounds already freed from microcomponents that can retain them can be a key to the formulation of functional foods.

## AUTHOR CONTRIBUTIONS

**Giulia Basile**: Conceptualization; investigation; writing—original draft; data curation. **Lucia De Luca**: Conceptualization; writing—review and editing; investigation; methodology; visualization. **Giovanni Sorrentino**: Data curation; investigation; writing—original draft. **Martina Calabrese**: Data curation; investigation; writing—original draft. **Mariarca Esposito**: Investigation; data curation; writing—original draft. **Fabiana Pizzolongo**: Investigation. **Raffaele Romano**: Conceptualization; visualization; supervision; resources; methodology.

## CONFLICT OF INTEREST STATEMENT

The authors declare no conflicts of interest.

## References

[jfds17487-bib-0001] Abdelrahman, R. , Hamdi, M. , Baba, W. N. , Hassan, H. M. , & Maqsood, S. (2023). Synergistic combination of natural deep eutectic solvents and green extraction techniques for the valorization of date palm leaves: Optimization of the solvent‐biomass interaction. Microchemical Journal, 195, 109503. 10.1016/j.microc.2023.109503

[jfds17487-bib-0002] Ahangari, H. , King, J. W. , Ehsani, A. , & Yousefi, M. (2021). Supercritical fluid extraction of seed oils—A short review of current trends. Trends in Food Science & Technology, 111, 249–260. 10.1016/j.tifs.2021.02.066

[jfds17487-bib-0003] Aiello, F. , Restuccia, D. , Spizzirri, U. G. , Carullo, G. , Leporini, M. , & Loizzo, M. R. (2020). Improving kefir bioactive properties by functional enrichment with plant and agro‐food waste extracts. Fermentation, 6(3), 83. 10.3390/fermentation6030083

[jfds17487-bib-0004] Akgün, N. A. , Bulut, H. , Kikic, I. , & Solinas, D. (2014). Extraction behavior of lipids obtained from spent coffee grounds using supercritical carbon dioxide. Chemical Engineering & Technology, 37(11), 1975–1981. 10.1002/ceat.201400237

[jfds17487-bib-0005] Al Khawli, F. , Pateiro, M. , Domínguez, R. , Lorenzo, J. M. , Gullón, P. , Kousoulaki, K. , Ferrer, E. , Berrada, H. , & Barba, F. J. (2019). Innovative green technologies of intensification for valorization of seafood and their by‐products. Marine Drugs, 17(12), 689. 10.3390/md17120689 31817754 PMC6950251

[jfds17487-bib-0006] Al‐Ghouti, M. A. , Khan, M. , Nasser, M. S. , Al Saad, K. , & Heng, O. E. (2021). A novel method for metals extraction from municipal solid waste using a microwave‐assisted acid extraction. Journal of Cleaner Production, 287, 125039. 10.1016/j.jclepro.2020.125039

[jfds17487-bib-0007] Al‐Hindi, R. R. , & Abd El Ghani, S. (2020). Production of functional fermented milk beverages supplemented with pomegranate peel extract and probiotic lactic acid bacteria. Journal of Food Quality, 2020, 1–9. 10.1155/2020/4710273

[jfds17487-bib-0008] Aliakbarian, B. , Casale, M. , Paini, M. , Casazza, A. A. , Lanteri, S. , & Perego, P. (2015). Production of a novel fermented milk fortified with natural antioxidants and its analysis by NIR spectroscopy. LWT—Food Science and Technology, 62(1, Part 2), 376–383. 10.1016/j.lwt.2014.07.037

[jfds17487-bib-0009] AlYammahi, J. , Rambabu, K. , Thanigaivelan, A. , Bharath, G. , Hasan, S. W. , Show, P. L. , & Banat, F. (2023). Advances of non‐conventional green technologies for phyto‐saccharides extraction: Current status and future perspectives. Phytochemistry Reviews, 22(4), 1067–1088. 10.1007/s11101-022-09831-2

[jfds17487-bib-0010] Araújo, M. N. , Azevedo, A. Q. P. L. , Hamerski, F. , Voll, F. A. P. , & Corazza, M. L. (2019). Enhanced extraction of spent coffee grounds oil using high‐pressure CO_2_ plus ethanol solvents. Industrial Crops and Products, 141, 111723. 10.1016/j.indcrop.2019.111723

[jfds17487-bib-0011] Argenziano, R. , Moccia, F. , Esposito, R. , D'Errico, G. , Panzella, L. , & Napolitano, A. (2022). Recovery of lignins with potent antioxidant properties from shells of edible nuts by a green ball milling/deep eutectic solvent (des)‐based protocol. Antioxidants, 11(10), 1860. 10.3390/antiox11101860 36290583 PMC9598286

[jfds17487-bib-0012] Argun, M. E. , Argun, M. Ş. , Arslan, F. N. , Nas, B. , Ates, H. , Tongur, S. , & Cakmakcı, O. (2022). Recovery of valuable compounds from orange processing wastes using supercritical carbon dioxide extraction. Journal of Cleaner Production, 375, 134169. 10.1016/j.jclepro.2022.134169

[jfds17487-bib-0013] Azuan, A. A. , Mohd Zin, Z. , Hasmadi, M. , Rusli, N. D. , & Zainol, M. K. (2020). Physicochemical, antioxidant and sensory characteristics of cookies supplemented with different levels of spent coffee ground extract. Food Research, 4(4), 1181–1190. 10.26656/fr.2017.4(4).058

[jfds17487-bib-0014] Báez, J. , Fernández‐Fernández, A. M. , Briozzo, F. , Díaz, S. , Dorgans, A. , Tajam, V. , & Medrano, A. (2021). Effect of enzymatic hydrolysis of Brewer's spent grain on bioactivity, techno‐functional properties, and nutritional value when added to a bread formulation. Biology and Life Sciences Forum, 6(1), 100. 10.3390/Foods2021-11024

[jfds17487-bib-0015] Banihani, S. , Swedan, S. , & Alguraan, Z. (2013). Pomegranate and type 2 diabetes. Nutrition Research, 33(5), 341–348. 10.1016/j.nutres.2013.03.003 23684435

[jfds17487-bib-0016] Barbera, M. (2020). Reuse of food waste and wastewater as a source of polyphenolic compounds to use as food additives. Journal of AOAC International, 103(4), 906–914. 10.1093/jaocint/qsz025 33241336

[jfds17487-bib-0017] Bertolo, M. R. V. , Martins, V. C. A. , Plepis, A. M. G. , & Bogusz, S. (2021). Utilization of pomegranate peel waste: Natural deep eutectic solvents as a green strategy to recover valuable phenolic compounds. Journal of Cleaner Production, 327, 129471. 10.1016/j.jclepro.2021.129471

[jfds17487-bib-0018] Biel‐Nielsen, T. L. , Li, K. , Sørensen, S. O. , Sejberg, J. J. P. , Meyer, A. S. , & Holck, J. (2022). Utilization of industrial citrus pectin side streams for enzymatic production of human milk oligosaccharides. Carbohydrate Research, 519, 108627. 10.1016/j.carres.2022.108627 35803019

[jfds17487-bib-0019] Catalkaya, G. , & Kahveci, D. (2019). Optimization of enzyme assisted extraction of lycopene from industrial tomato waste. Separation and Purification Technology, 219, 55–63. 10.1016/j.seppur.2019.03.006

[jfds17487-bib-0020] Chedea, V. S. , Palade, L. M. , Marin, D. E. , Pelmus, R. S. , Habeanu, M. , Rotar, M. C. , Gras, M. A. , Pistol, G. C. , & Taranu, I. (2018). Intestinal absorption and antioxidant activity of grape pomace polyphenols. Nutrients, 10(5), 588. 10.3390/nu10050588 29747456 PMC5986468

[jfds17487-bib-0021] Chemat, F. , Vian, M. A. , & Cravotto, G. (2012). Green extraction of natural products: Concept and principles. International Journal of Molecular Sciences, 13(7), 8615–8627. 10.3390/ijms13078615 22942724 PMC3430255

[jfds17487-bib-0022] Coelho, J. P. , Robalo, M. P. , Boyadzhieva, S. , & Stateva, R. P. (2021). Microwave‐assisted extraction of phenolic compounds from spent coffee grounds. Process optimization applying design of experiments. Molecules, 26(23), 7320. 10.3390/molecules26237320 34885901 PMC8658841

[jfds17487-bib-0023] Cremonini, F. , Di Caro, S. , Nista, E. C. , Bartolozzi, F. , Capelli, G. , Gasbarrini, G. , & Gasbarrini, A. (2002). Meta‐analysis: The effect of probiotic administration on antibiotic‐associated diarrhoea. Alimentary Pharmacology & Therapeutics, 16(8), 1461–1467. 10.1046/j.1365-2036.2002.01318.x 12182746

[jfds17487-bib-0024] Cukrowska, B. , Motyl, I. , Kozáková, H. , Schwarzer, M. , Górecki, R. K. , Klewicka, E. , Śliżewska, K. , & Libudzisz, Z. (2009). Probiotic Lactobacillus strains: In vitro and in vivo studies. Folia Microbiologica, 54(6), 533–537. 10.1007/s12223-009-0077-7 20140722

[jfds17487-bib-0025] Dahmoune, F. , Boulekbache, L. , Moussi, K. , Aoun, O. , Spigno, G. , & Madani, K. (2013). Valorization of *Citrus limon* residues for the recovery of antioxidants: Evaluation and optimization of microwave and ultrasound application to solvent extraction. Industrial Crops and Products, 50, 77–87. 10.1016/j.indcrop.2013.07.013

[jfds17487-bib-0026] Dauber, C. , Romero, M. , Chaparro, C. , Ureta, C. , Ferrari, C. , Lans, R. , Frugoni, L. , Echeverry, M. V. , Calvo, B. S. , Trostchansky, A. , Miraballes, M. , Gámbaro, A. , & Vieitez, I. (2024). Cookies enriched with coffee silverskin powder and coffee silverskin ultrasound extract to enhance fiber content and antioxidant properties. Applied Food Research, 4(1), 100373. 10.1016/j.afres.2023.100373

[jfds17487-bib-0027] De Vries, J. A. , den Uijl, C. H. , Voragen, A. G. J. , Rombouts, F. M. , & Pilnik, W. (1983). Structural features of the neutral sugar side chains of apple pectic substances. Carbohydrate Polymers, 3(3), 193–205. 10.1016/0144-8617(83)90018-8

[jfds17487-bib-0028] De Vries, J. A. , Rombouts, F. M. , Voragen, A. G. J. , & Pilnik, W. (1982). Enzymic degradation of apple pectins. Carbohydrate Polymers, 2(1), 25–33. 10.1016/0144-8617(82)90043-1

[jfds17487-bib-0029] De Vries, J. A. , Voragen, A. G. J. , Rombouts, F. M. , & Pilnik, W. (1981). Extraction and purification of pectins from alcohol insoluble solids from ripe and unripe apples. Carbohydrate Polymers, 1(2), 117–127. 10.1016/0144-8617(81)90004-7

[jfds17487-bib-0030] Deng, Q. , Penner, M. H. , & Zhao, Y. (2011). Chemical composition of dietary fiber and polyphenols of five different varieties of wine grape pomace skins. Food Research International, 44(9), 2712–2720. 10.1016/j.foodres.2011.05.026

[jfds17487-bib-0031] Dimou, C. , & Koutelidakis, A. (2016). Value added alternatives of winemaking process residues: A health based oriented perspective. BAOJ Biotechnology, 2, 016.

[jfds17487-bib-0032] Doria, E. , Boncompagni, E. , Marra, A. , Dossena, M. , Verri, M. , & Buonocore, D. (2021). Polyphenols extraction from vegetable wastes using a green and sustainable method. Frontiers in Sustainable Food Systems, 5, 1–7. 10.3389/fsufs.2021.690399

[jfds17487-bib-0033] Ekezie, F.‐G. C. , Sun, D.‐W. , & Cheng, J.‐H. (2017). Acceleration of microwave‐assisted extraction processes of food components by integrating technologies and applying emerging solvents: A review of latest developments. Trends in Food Science & Technology, 67, 160–172. 10.1016/j.tifs.2017.06.006

[jfds17487-bib-0034] El Barnossi, A. , Moussaid, F. , & Housseini, A. I. (2021). Tangerine, banana and pomegranate peels valorisation for sustainable environment: A review. Biotechnology Reports, 29, e00574. 10.1016/j.btre.2020.e00574 33376681 PMC7758358

[jfds17487-bib-0035] El Kantar, S. , Rajha, H. N. , Boussetta, N. , Vorobiev, E. , Maroun, R. G. , & Louka, N. (2019). Green extraction of polyphenols from grapefruit peels using high voltage electrical discharges, deep eutectic solvents and aqueous glycerol. Food Chemistry, 295, 165–171. 10.1016/j.foodchem.2019.05.111 31174746

[jfds17487-bib-0036] Eller, F. J. , Moser, J. K. , Kenar, J. A. , & Taylor, S. L. (2010). Extraction and analysis of tomato seed oil. Journal of the American Oil Chemists’ Society, 87(7), 755–762. 10.1007/s11746-010-1563-4

[jfds17487-bib-0037] Fernández, M. , de los, Á. , Espino, M. , Gomez, F. J. V. , & Silva, M. F. (2018). Novel approaches mediated by tailor‐made green solvents for the extraction of phenolic compounds from agro‐food industrial by‐products. Food Chemistry, 239, 671–678. 10.1016/j.foodchem.2017.06.150 28873620

[jfds17487-bib-0038] Fu, C. , Tian, H. , Li, Q. , Cai, T. , & Du, W. (2006). Ultrasound‐assisted extraction of xyloglucan from apple pomace. Ultrasonics Sonochemistry, 13(6), 511–516. 10.1016/j.ultsonch.2005.09.007 16325452

[jfds17487-bib-0039] Garcia‐Castello, E. M. , Rodriguez‐Lopez, A. D. , Mayor, L. , Ballesteros, R. , Conidi, C. , & Cassano, A. (2015). Optimization of conventional and ultrasound assisted extraction of flavonoids from grapefruit (*Citrus paradisi* L.) solid wastes. LWT—Food Science and Technology, 64(2), 1114–1122. 10.1016/j.lwt.2015.07.024

[jfds17487-bib-0040] García‐Roldán, A. , Piriou, L. , & Jauregi, P. (2023). Natural deep eutectic solvents as a green extraction of polyphenols from spent coffee grounds with enhanced bioactivities. Frontiers in Plant Science, 13, 1072592. 10.3389/fpls.2022.1072592 36714731 PMC9874221

[jfds17487-bib-0041] Gardiner, G. E. , Bouchier, P. , O'Sullivan, E. , Kelly, J. , Kevin Collins, J. , Fitzgerald, G. , Paul Ross, R. , & Stanton, C. (2002). A spray‐dried culture for probiotic Cheddar cheese manufacture. International Dairy Journal, 12(9), 749–756. 10.1016/S0958-6946(02)00072-9

[jfds17487-bib-0042] Garnett, M. T. , Kumar, H. K. S. , Beckingham, B. S. , & Alexander, S. L. (2024). Extraction of cellulose from restaurant food waste. RSC Sustainability, 2(1), 170–178. 10.1039/d3su00192j

[jfds17487-bib-0043] Gavahian, M. , Mathad, G. N. , Pandiselvam, R. , Lin, J. , & Sun, D.‐W. (2021). Emerging technologies to obtain pectin from food processing by‐products: A strategy for enhancing resource efficiency. Trends in Food Science & Technology, 115, 42–54. 10.1016/j.tifs.2021.06.018

[jfds17487-bib-0044] George, B. , Kaur, C. , Khurdiya, D. S. , & Kapoor, H. C. (2004). Antioxidants in tomato (*Lycopersium esculentum*) as a function of genotype. Food Chemistry, 84(1), 45–51. 10.1016/S0308-8146(03)00165-1

[jfds17487-bib-0045] Georgiev, R. , Kalaydzhiev, H. , Slavov, A. , Ivanova, P. , Uzunova, G. , & Chalova, V. I. (2022). Residual waste after protein isolation from ethanol‐treated rapeseed meal has physico‐chemical properties for functional food systems formulation. Waste and Biomass Valorization, 13(2), 1223–1232. 10.1007/s12649-021-01567-y

[jfds17487-bib-0046] Gobbetti, M. , Cagno, R. D. , & De Angelis, M. (2010). Functional microorganisms for functional food quality. Critical Reviews in Food Science and Nutrition, 50(8), 716–727. 10.1080/10408398.2010.499770 20830633

[jfds17487-bib-0047] Gómez‐Mejía, E. , Rosales‐Conrado, N. , León‐González, M. E. , & Madrid, Y. (2019). Citrus peels waste as a source of value‐added compounds: Extraction and quantification of bioactive polyphenols. Food Chemistry, 295, 289–299. 10.1016/j.foodchem.2019.05.136 31174761

[jfds17487-bib-0048] Granato, D. , Barba, F. J. , Bursać Kovačević, D. , Lorenzo, J. M. , Cruz, A. G. , & Putnik, P. (2020). Functional foods: Product development, technological trends, efficacy testing, and safety. Annual Review of Food Science and Technology, 11(1), 93–118. 10.1146/annurev-food-032519-051708 31905019

[jfds17487-bib-0049] Grassino, A. N. , Brnčić, M. , Vikić‐Topić, D. , Roca, S. , Dent, M. , & Brnčić, S. R. (2016). Ultrasound assisted extraction and characterization of pectin from tomato waste. Food Chemistry, 198, 93–100. 10.1016/j.foodchem.2015.11.095 26769509

[jfds17487-bib-0050] Guo, Q. , Sun, D. W. , Cheng, J. H. , & Han, Z. (2017). Microwave processing techniques and their recent applications in the food industry. Trends in Food Science & Technology, 67, 236–247. 10.1016/j.tifs.2017.07.007

[jfds17487-bib-0051] Guo, R. , Lv, S. , Liao, T. , Xi, F. , Zhang, J. , Zuo, X. , Cao, X. , Feng, Z. , & Zhang, Y. (2020). Classifying green technologies for sustainable innovation and investment. Resources, Conservation and Recycling, 153, 104580. 10.1016/j.resconrec.2019.104580

[jfds17487-bib-0052] Husanu, E. , Mero, A. , Rivera, J. G. , Mezzetta, A. , Ruiz, J. C. , D'Andrea, F. , Pomelli, S. C. , & Guazzelli, L. (2020). Exploiting deep eutectic solvents and ionic liquids for the valorization of chestnut shell waste. ACS Sustainable Chemistry & Engineering, 8(50), 18386–18399. 10.1021/acssuschemeng.0c04945?ref=pdf

[jfds17487-bib-0053] Ignat, I. , Volf, I. , & Popa, V. I. (2011). A critical review of methods for characterisation of polyphenolic compounds in fruits and vegetables. Food Chemistry, 126(4), 1821–1835. 10.1016/j.foodchem.2010.12.026 25213963

[jfds17487-bib-0054] Imeneo, V. , Romeo, R. , Gattuso, A. , De Bruno, A. , & Piscopo, A. (2021). Functionalized biscuits with bioactive ingredients obtained by Citrus lemon pomace. Foods, 10(10), 2460. 10.3390/foods10102460 34681509 PMC8536132

[jfds17487-bib-0055] Iriondo‐DeHond, M. , Blázquez‐Duff, J. M. , del Castillo, M. D. , & Miguel, E. (2020). Nutritional quality, sensory analysis and shelf life stability of yogurts containing inulin‐type fructans and winery byproducts for sustainable health. Foods, 9(9), 1199. 10.3390/foods9091199 32878017 PMC7554681

[jfds17487-bib-0056] Jokić, S. , Molnar, M. , Cikoš, A. M. , Jakovljević, M. , Šafranko, S. , & Jerković, I. (2020). Separation of selected bioactive compounds from orange peel using the sequence of supercritical CO_2_ extraction and ultrasound solvent extraction: Optimization of limonene and hesperidin content. Separation Science and Technology, 55(15), 2799–2811. 10.1080/01496395.2019.1647245

[jfds17487-bib-0057] Kaderides, K. , Papaoikonomou, L. , Serafim, M. , & Goula, A. M. (2019). Microwave‐assisted extraction of phenolics from pomegranate peels: Optimization, kinetics, and comparison with ultrasounds extraction. Chemical Engineering and Processing‐Process Intensification, 137, 1–11. 10.1016/j.cep.2019.01.006

[jfds17487-bib-0058] Kan, L. , Oliviero, T. , Verkerk, R. , Fogliano, V. , & Capuano, E. (2020). Interaction of bread and berry polyphenols affects starch digestibility and polyphenols bio‐accessibility. Journal of Functional Foods, 68, 103924. 10.1016/j.jff.2020.103924

[jfds17487-bib-0059] Karbuz, P. , & Tugrul, N. (2021). Microwave and ultrasound assisted extraction of pectin from various fruits peel. Journal of Food Science and Technology, 58(2), 641–650. 10.1007/s13197-020-04578-0 33568858 PMC7847832

[jfds17487-bib-0060] Kaur, S. , Panesar, P. S. , & Chopra, H. K. (2023). Extraction of dietary fiber from Kinnow (*Citrus reticulata*) peels using sequential ultrasonic and enzymatic treatments and its application in development of cookies. Food Bioscience, 54, 102891. 10.1016/j.fbio.2023.102891

[jfds17487-bib-0062] Kertesz, Z. I. (1951). The pectic substances. Interscience Publishers.

[jfds17487-bib-0063] Kim, Y. , Keogh, J. B. , & Clifton, P. M. (2016). Polyphenols and glycemic control. Nutrients, 8(1), 17. 10.3390/nu8010017 26742071 PMC4728631

[jfds17487-bib-0064] Koksel, H. , Tekin‐Cakmak, Z. H. , Oruc, S. , Kilic, G. , Ozkan, K. , Cetiner, B. , Sagdic, O. , Sestili, F. , & Jilal, A. (2024). A new functional wheat flour flatbread (Bazlama) enriched with high‐β‐glucan hull‐less barley flour. Foods, 13(2), 326. 10.3390/foods13020326 38275693 PMC10814883

[jfds17487-bib-0065] Kumcuoglu, S. , Yilmaz, T. , & Tavman, S. (2014). Ultrasound assisted extraction of lycopene from tomato processing wastes. Journal of Food Science and Technology, 51, 4102–4107. 10.1007/s13197-013-0926-x 25477688 PMC4252437

[jfds17487-bib-0066] Lameirão, F. , Pinto, D. , Vieira, E. F. , Peixoto, A. F. , Freire, C. , Sut, S. , Dell'Acqua, S. , Costa, P. , Delerue‐Matos, C. , & Rodrigues, F. (2020). Green‐sustainable recovery of phenolic and antioxidant compounds from industrial chestnut shells using ultrasound‐assisted extraction: Optimization and evaluation of biological activities in vitro. Antioxidants, 9(3), 267. 10.3390/antiox9030267 32213812 PMC7139998

[jfds17487-bib-0067] Lamsal, B. P. , & Faubion, J. M. (2009). The beneficial use of cereal and cereal components in probiotic foods. Food Reviews International, 25(2), 103–114. 10.1080/87559120802682573

[jfds17487-bib-0068] Lanjekar, K. J. , & Rathod, V. K. (2021). Green extraction of glycyrrhizic acid from *Glycyrrhiza glabra* using choline chloride based natural deep eutectic solvents (NADESs). Process Biochemistry, 102, 22–32. 10.1016/j.procbio.2020.11.023

[jfds17487-bib-0069] Lasunon, P. , & Sengkhamparn, N. (2022). Effect of ultrasound‐assisted, microwave‐assisted and ultrasound‐microwave‐assisted extraction on pectin extraction from industrial tomato waste. Molecules, 27(4), 1157. 10.3390/molecules27041157 35208946 PMC8877420

[jfds17487-bib-0070] Lasunon, P. , Phonkerd, N. , Tettawong, P. , & Sengkhamparn, N. (2021). Effect of microwave‐assisted extraction on bioactive compounds from industrial tomato waste and its antioxidant activity. Food Research, 5(2), 468–474. 10.26656/fr.2017.5(2).516

[jfds17487-bib-0071] Lavelli, V. , & Corti, S. (2011). Phloridzin and other phytochemicals in apple pomace: Stability evaluation upon dehydration and storage of dried product. Food Chemistry, 129(4), 1578–1583. 10.1016/j.foodchem.2011.06.011

[jfds17487-bib-0072] Li, W. , Yang, R. , Ying, D. , Yu, J. , Sanguansri, L. , & Augustin, M. A. (2020). Analysis of polyphenols in apple pomace: A comparative study of different extraction and hydrolysis procedures. Industrial Crops and Products, 147, 112250. 10.1016/j.indcrop.2020.112250

[jfds17487-bib-0073] Li, Y. , He, D. , Li, B. , Lund, M. N. , Xing, Y. , Wang, Y. , Li, F. , Cao, X. , Liu, Y. , Chen, X. , Yu, J. , Zhu, J. , Zhang, M. , Wang, Q. , Zhang, Y. , Li, B. , Wang, J. , Xing, X. , & Li, L. (2021). Engineering polyphenols with biological functions via polyphenol‐protein interactions as additives for functional foods. Trends in Food Science & Technology, 110, 470–482. 10.1016/j.tifs.2021.02.009

[jfds17487-bib-0074] Li, Y. , Yuan, F. , Wu, Y. , Zhang, Y. , Gao, B. , & Yu, L. (2020). Triacylglycerols and fatty acid compositions of cucumber, tomato, pumpkin, and carrot seed oils by ultra‐performance convergence chromatography combined with quadrupole time‐of‐flight mass spectrometry. Foods, 9(8), 970. 10.3390/foods9080970 32707916 PMC7466086

[jfds17487-bib-0075] Liew, S. Q. , Ngoh, G. C. , Yusoff, R. , & Teoh, W. H. (2018). Acid and deep eutectic solvent (DES) extraction of pectin from pomelo (*Citrus grandis* (L.) Osbeck) peels. Biocatalysis and Agricultural Biotechnology, 13, 1–11. 10.1016/j.bcab.2017.11.001

[jfds17487-bib-0076] Lin, S. (2022). Dietary fiber in bakery products: Source, processing, and function. Advances in Food and Nutrition Research, 99, 37–100. 10.1016/bs.afnr.2021.12.001 35595397

[jfds17487-bib-0077] Liu, W. , Zhang, K. , Chen, J. , & Yu, J. (2018). Ascorbic acid and choline chloride: A new natural deep eutectic solvent for extracting *tert*‐butylhydroquinone antioxidant. Journal of Molecular Liquids, 260, 173–179. 10.1016/j.molliq.2018.03.092

[jfds17487-bib-0078] Liu, X. , Le Bourvellec, C. , & Renard, C. M. G. C. (2020). Interactions between cell wall polysaccharides and polyphenols: Effect of molecular internal structure. Comprehensive Reviews in Food Science and Food Safety, 19(6), 3574–3617. 10.1111/1541-4337.12632 33337054

[jfds17487-bib-0079] Lombardelli, C. , Benucci, I. , Mazzocchi, C. , & Esti, M. (2021). A novel process for the recovery of Betalains from unsold red beets by low‐temperature enzyme‐assisted extraction. Foods, 10(2), 236. 10.3390/foods10020236 33498835 PMC7911046

[jfds17487-bib-0080] Maimulyanti, A. , Nurhidayati, I. , Mellisani, B. , Putri, F. A. R. , Puspita, F. , & Prihadi, A. R. (2023). Development of natural deep eutectic solvent (NADES) based on choline chloride as a green solvent to extract phenolic compound from coffee husk waste. Arabian Journal of Chemistry, 16(4), 104634. 10.1016/j.arabjc.2023.104634

[jfds17487-bib-0081] Mansour, M. S. M. , Abdel‐Shafy, H. I. , & Mehaya, F. M. S. (2018). Valorization of food solid waste by recovery of polyphenols using hybrid molecular imprinted membrane. Journal of Environmental Chemical Engineering, 6(4), 4160–4170. 10.1016/j.jece.2018.06.019

[jfds17487-bib-0082] Marić, M. , Grassino, A. N. , Zhu, Z. , Barba, F. J. , Brnčić, M. , & Rimac Brnčić, S. (2018). An overview of the traditional and innovative approaches for pectin extraction from plant food wastes and by‐products: Ultrasound‐, microwaves‐, and enzyme‐assisted extraction. Trends in Food Science & Technology, 76, 28–37. 10.1016/j.tifs.2018.03.022

[jfds17487-bib-0083] Martins, Z. E. , Pinho, O. , & Ferreira, I. M. P. L. V. O. (2017). Food industry by‐products used as functional ingredients of bakery products. Trends in Food Science & Technology, 67, 106–128. 10.1016/j.tifs.2017.07.003

[jfds17487-bib-0084] Meerasri, J. , & Sothornvit, R. (2022). Novel development of coffee oil extracted from spent coffee grounds as a butter substitute in bakery products. Journal of Food Processing and Preservation, 46(7), e16687. 10.1111/jfpp.16687

[jfds17487-bib-0085] Min, B. , Lim, J. , Ko, S. , Lee, K.‐G. , Lee, S. H. , & Lee, S. (2011). Environmentally friendly preparation of pectins from agricultural byproducts and their structural/rheological characterization. Bioresource Technology, 102(4), 3855–3860. 10.1016/j.biortech.2010.12.019 21193307

[jfds17487-bib-0086] Minjares‐Fuentes, R. , Femenia, A. , Garau, M. C. , Meza‐Velázquez, J. A. , Simal, S. , & Rosselló, C. (2014). Ultrasound‐assisted extraction of pectins from grape pomace using citric acid: A response surface methodology approach. Carbohydrate Polymers, 106, 179–189. 10.1016/j.carbpol.2014.02.013 24721067

[jfds17487-bib-0087] Mitelut, A. C. , Popa, E. E. , Popescu, P. A. , & Popa, M. E. (2021). Trends of innovation in bread and bakery production. In C. Galanakis (Ed.), Trends in wheat and bread making (1st ed., pp. 199–226). Academic Press. 10.1016/B978-0-12-821048-2.00007-6

[jfds17487-bib-0088] Moccia, F. , Gallucci, N. , Giovando, S. , Zuorro, A. , Lavecchia, R. , D'Errico, G. , Panzella, L. , & Napolitano, A. (2022). A tunable deep eutectic solvent‐based processing for valorization of chestnut wood fiber as a source of ellagic acid and lignin. Journal of Environmental Chemical Engineering, 10(3), 107773. 10.1016/j.jece.2022.107773

[jfds17487-bib-0089] Morcelli, A. , Cassel, E. , Vargas, R. , Rech, R. , & Marcílio, N. (2021). Supercritical fluid (CO_2_+ ethanol) extraction of chlorophylls and carotenoids from Chlorella sorokiniana: COSMO‐SAC assisted prediction of properties and experimental approach. Journal of CO Utilization, 51, 101649. 10.1016/j.jcou.2021.101649

[jfds17487-bib-0090] Muangrat, R. , & Pongsirikul, I. (2019). Recovery of spent coffee grounds oil using supercritical CO_2_: Extraction optimisation and physicochemical properties of oil. CyTA—Journal of Food, 17(1), 334–346. 10.1080/19476337.2019.1580771

[jfds17487-bib-0091] Munekata, P. E. S. , Pateiro, M. , Zhang, W. , Dominguez, R. , Xing, L. , Fierro, E. M. , & Lorenzo, J. M. (2021). Health benefits, extraction and development of functional foods with curcuminoids. Journal of Functional Foods, 79, 104392. 10.1016/j.jff.2021.104392

[jfds17487-bib-0092] Mushtaq, M. , Sultana, B. , Anwar, F. , Adnan, A. , & Rizvi, S. S. (2015). Enzyme‐assisted supercritical fluid extraction of phenolic antioxidants from pomegranate peel. The Journal of Supercritical Fluids, 104, 122–131. 10.1016/j.supflu.2015.05.020

[jfds17487-bib-0093] Nicolosi, A. , Laganà, V. R. , & Di Gregorio, D. (2023). Habits, health and environment in the purchase of bakery products: Consumption preferences and sustainable inclinations before and during COVID‐19. Foods, 12(8), 1661. 10.3390/foods12081661 37107456 PMC10138246

[jfds17487-bib-0139] Othman, S. , Añibarro‐Ortega, M. , Dias, M. I. , Ćirić, A. , Mandim, F. , Soković, M. , Ferreira, C. F. R. I. , Pinela, J. , & Barros, L. (2022). Valorization of quince peel into functional food ingredients: A path towards “zero waste” and sustainable food systems. Heliyon, 8(10). 10.1016/j.heliyon.2022.e11042 PMC9587281 36281371

[jfds17487-bib-0094] Panić, M. , Andlar, M. , Tišma, M. , Rezić, T. , Šibalić, D. , Cvjetko Bubalo, M. , & Radojčić Redovniković, I. (2021). Natural deep eutectic solvent as a unique solvent for valorisation of orange peel waste by the integrated biorefinery approach. Waste Management, 120, 340–350. 10.1016/j.wasman.2020.11.052 33340816

[jfds17487-bib-0095] Panzella, L. , Moccia, F. , Nasti, R. , Marzorati, S. , Verotta, L. , & Napolitano, A. (2020). Bioactive phenolic compounds from agri‐food wastes: An update on green and sustainable extraction methodologies. Frontiers in Nutrition, 7, 60. 10.3389/fnut.2020.00060 32457916 PMC7221145

[jfds17487-bib-0096] Petrotos, K. , Giavasis, I. , Gerasopoulos, K. , Mitsagga, C. , Papaioannou, C. , & Gkoutsidis, P. (2021). Optimization of the vacuum microwave assisted extraction of the natural polyphenols and flavonoids from the raw solid waste of the pomegranate juice producing industry at industrial scale. Molecules, 26(4), 1033. 10.3390/molecules26041033 33669172 PMC7919679

[jfds17487-bib-0097] Popovic, B. M. , Micic, N. , Potkonjak, A. , Blagojevic, B. , Pavlovic, K. , Milanov, D. , & Juric, T. (2022). Novel extraction of polyphenols from sour cherry pomace using natural deep eutectic solvents—Ultrafast microwave‐assisted NADES preparation and extraction. Food Chemistry, 366, 130562. 10.1016/j.foodchem.2021.130562 34289442

[jfds17487-bib-0098] Prakash Maran, J. , Sivakumar, V. , Thirugnanasambandham, K. , & Sridhar, R. (2013). Optimization of microwave assisted extraction of pectin from orange peel. Carbohydrate Polymers, 97(2), 703–709. 10.1016/j.carbpol.2013.05.052 23911504

[jfds17487-bib-0099] Qian, M. , Liu, D. , Zhang, X. , Yin, Z. , Ismail, B. B. , Ye, X. , & Guo, M. (2021). A review of active packaging in bakery products: Applications and future trends. Trends in Food Science & Technology, 114, 459–471. 10.1016/j.tifs.2021.06.009

[jfds17487-bib-0100] Quan, N. H. K. , Yen, N. T. N. , & Chung, D. D. (2020). Functional food in Viet Nam: Trends consumer online shopping in Ho Chi Minh city. IOP Conference Series: Materials Science and Engineering, 991(1), 012037. 10.1088/1757-899X/991/1/012037

[jfds17487-bib-0101] Radošević, K. , Ćurko, N. , Gaurina Srček, V. , Cvjetko Bubalo, M. , Tomašević, M. , Kovačević Ganić, K. , & Radojčić Redovniković, I. (2016). Natural deep eutectic solvents as beneficial extractants for enhancement of plant extracts bioactivity. LWT, 73, 45–51. 10.1016/j.lwt.2016.05.037

[jfds17487-bib-0102] Rašić, J. L. (2003). Microflora of the intestine | Probiotics. In B. Caballero (Eds.), Encyclopedia of food sciences and nutrition (2nd Ed, pp. 3911–3916). Academic Press. 10.1016/B0-12-227055-X/00776-8

[jfds17487-bib-0103] Raveendran, S. , Parameswaran, B. , Ummalyma, S. B. , Abraham, A. , Mathew, A. K. , Madhavan, A. , Rebello, S. , & Pandey, A. (2018). Applications of microbial enzymes in food industry. Food Technology and Biotechnology, 56(1), 16. 10.17113/ftb.56.01.18.5491 29795993 PMC5956270

[jfds17487-bib-0104] Rezzadori, K. , Benedetti, S. , & Amante, E. R. (2012). Proposals for the residues recovery: Orange waste as raw material for new products. Food and Bioproducts Processing, 90(4), 606–614. 10.1016/j.fbp.2012.06.002

[jfds17487-bib-0105] Roberfroid, M. B. (2002). Global view on functional foods: European perspectives. British Journal of Nutrition, 88(S2), S133–S138. 10.1079/BJN2002677 12495454

[jfds17487-bib-0106] Romano, R. , Aiello, A. , Pizzolongo, F. , Rispoli, A. , De Luca, L. , & Masi, P. (2020). Characterisation of oleoresins extracted from tomato waste by liquid and supercritical carbon dioxide. International Journal of Food Science & Technology, 55(10), 3334–3342. 10.1111/ijfs.14597

[jfds17487-bib-0107] Romano, R. , De Luca, L. , Basile, G. , Nitride, C. , Pizzolongo, F. , & Masi, P. (2023). The use of carbon dioxide as a green approach to recover bioactive compounds from spent coffee grounds. Foods, 12(10), 1958. 10.3390/foods12101958 37238777 PMC10217628

[jfds17487-bib-0108] Ropartz, D. , & Ralet, M.‐C. (2020). Pectin structure. In V. Kontogiorgos (Eds.), Pectin: Technological and physiological properties (pp. 17–36). Springer International Publishing. 10.1007/978-3-030-53421-9_2

[jfds17487-bib-0109] Ruggieri, L. , Cadena, E. , Martínez‐Blanco, J. , Gasol, C. M. , Rieradevall, J. , Gabarrell, X. , Gea, T. , Sort, X. , & Sánchez, A. (2009). Recovery of organic wastes in the Spanish wine industry. Technical, economic and environmental analyses of the composting process. Journal of Cleaner Production, 17(9), 830–838. 10.1016/j.jclepro.2008.12.005

[jfds17487-bib-0110] Rukavina, I. , Rodrigues, M. J. , Pereira, C. G. , Mansinhos, I. , Romano, A. , Ślusarczyk, S. , Matkowski, A. , & Custódio, L. (2021). Greener is better: First approach for the use of natural deep eutectic solvents (NADES) to extract antioxidants from the medicinal halophyte *Polygonum maritimum* L. Molecules, 26(20), 6136. 10.3390/molecules26206136 34684717 PMC8536975

[jfds17487-bib-0111] Salazar‐Bermeo, J. , Moreno‐Chamba, B. , Heredia‐Hortigüela, R. , Lizama, V. , Martínez‐Madrid, M. C. , Saura, D. , Valero, M. , Neacsu, M. , & Martí, N. (2023). Green technologies for persimmon by‐products revalorisation as sustainable sources of dietary fibre and antioxidants for functional beverages development. Antioxidants, 12(5), 1085. 10.3390/antiox12051085 37237951 PMC10215573

[jfds17487-bib-0112] Servili, M. , Rizzello, C. G. , Taticchi, A. , Esposto, S. , Urbani, S. , Mazzacane, F. , Di Maio, I. , Selvaggini, R. , Gobbetti, M. , & Di Cagno, R. (2011). Functional milk beverage fortified with phenolic compounds extracted from olive vegetation water, and fermented with functional lactic acid bacteria. International Journal of Food Microbiology, 147(1), 45–52. 10.1016/j.ijfoodmicro.2011.03.006 21458095

[jfds17487-bib-0113] Siró, I. , Kápolna, E. , Kápolna, B. , & Lugasi, A. (2008). Functional food. Product development, marketing and consumer acceptance—A review. Appetite, 51(3), 456–467. 10.1016/j.appet.2008.05.060 18582508

[jfds17487-bib-0114] Smolenski, K. (1923). Pectins. Rocznigi Chemki, 3, 86–152.

[jfds17487-bib-0115] Solaberrieta, I. , Mellinas, C. , Jiménez, A. , & Garrigós, M. C. (2022). Recovery of antioxidants from tomato seed industrial wastes by microwave‐assisted and ultrasound‐assisted extraction. Foods, 11(19), 3068. 10.3390/foods11193068 36230144 PMC9562903

[jfds17487-bib-0116] Squillace, P. , Adani, F. , & Scaglia, B. (2020). Supercritical CO_2_ extraction of tomato pomace: Evaluation of the solubility of lycopene in tomato oil as limiting factor of the process performance. Food Chemistry, 315, 126224. 10.1016/j.foodchem.2020.126224 32007813

[jfds17487-bib-0117] Stylianou, M. , Agapiou, A. , Omirou, M. , Vyrides, I. , Ioannides, I. M. , Maratheftis, G. , & Fasoula, D. (2018). Converting environmental risks to benefits by using spent coffee grounds (SCG) as a valuable resource. Environmental Science and Pollution Research, 25, 35776–35790. 10.1007/s11356-018-2359-6 29860699

[jfds17487-bib-0118] Sun‐Waterhouse, D. , & Wadhwa, S. S. (2013). Industry‐relevant approaches for minimising the bitterness of bioactive compounds in functional foods: A review. Food and Bioprocess Technology, 6(3), 607–627. 10.1007/s11947-012-0829-2

[jfds17487-bib-0119] Tabeshpour, J. , Hosseinzadeh, H. , Hashemzaei, M. , & Karimi, G. (2020). A review of the hepatoprotective effects of hesperidin, a flavanon glycoside in citrus fruits, against natural and chemical toxicities. DARU Journal of Pharmaceutical Sciences, 28(1), 305–317. 10.1007/s40199-020-00344-x 32277430 PMC7214587

[jfds17487-bib-0120] Taverna, M. J. (2018). Epidemiology of diabetes. In E. N. Cohen Sabban , F. M. Puchulu , & K. Cusi (Eds.), Dermatology and diabetes (pp. 1–6). Springer International Publishing. 10.1007/978-3-319-72475-1_1

[jfds17487-bib-0121] Toor, R. K. , & Savage, G. P. (2005). Antioxidant activity in different fractions of tomatoes. Food Research International, 38(5), 487–494. 10.1016/j.foodres.2004.10.016

[jfds17487-bib-0122] Tzani, A. , Kalafateli, S. , Tatsis, G. , Bairaktari, M. , Kostopoulou, I. , Pontillo, A. R. N. , & Detsi, A. (2021). Natural deep eutectic solvents (NaDESs) as alternative green extraction media for ginger (*Zingiber officinale* Roscoe). Sustainable Chemistry, 2(4), 576–598. 10.3390/suschem2040032

[jfds17487-bib-0123] Urbonavičienė, D. , Bobinaitė, R. , Trumbeckaitė, S. , Raudonė, L. , Janulis, V. , Bobinas, Č. , & Viškelis, P. (2018). Agro‐industrial tomato by‐products and extraction of functional food ingredients. Zemdirbyste‐Agriculture, 105(1), 63–70. 10.13080/z-a.2018.105.009

[jfds17487-bib-0124] Utekar, P. G. , Kininge, M. M. , & Gogate, P. R. (2021). Intensification of delignification and enzymatic hydrolysis of orange peel waste using ultrasound for enhanced fermentable sugar production. Chemical Engineering and Processing‐Process Intensification, 168, 108556. 10.1016/j.cep.2021.108556

[jfds17487-bib-0125] Uwineza, P. A. , & Waśkiewicz, A. (2020). Recent advances in supercritical fluid extraction of natural bioactive compounds from natural plant materials. Molecules, 25(17), 3847. 10.3390/molecules25173847 32847101 PMC7504334

[jfds17487-bib-0126] Vandeponseele, A. , Draye, M. , Piot, C. , Bernard, D. , Fanget, P. , & Chatel, G. (2022). Supercritical carbon dioxide in presence of water for the valorization of spent coffee grounds: Optimization by response surface methodology and investigation of caffeine extraction mechanism. Foods, 11(24), 4089. 10.3390/foods11244089 36553832 PMC9777831

[jfds17487-bib-0127] Vilas‐Franquesa, A. , Fryganas, C. , Casertano, M. , Montemurro, M. , & Fogliano, V. (2024). Upcycling mango peels into a functional ingredient by combining fermentation and enzymatic‐assisted extraction. Food Chemistry, 434, 137515. 10.1016/j.foodchem.2023.137515 37741240

[jfds17487-bib-0128] Vinatoru, M. , Mason, T. J. , & Calinescu, I. (2017). Ultrasonically assisted extraction (UAE) and microwave assisted extraction (MAE) of functional compounds from plant materials. TrAC Trends in Analytical Chemistry, 97, 159–178. 10.1016/j.trac.2017.09.002

[jfds17487-bib-0129] Vojvodić Cebin, A. , Šeremet, D. , Mandura, A. , Martinić, A. , & Komes, D. (2020). Onion solid waste as a potential source of functional food ingredients. Engineering Power : Bulletin of the Croatian Academy of Engineering, 15(3), 7–13.

[jfds17487-bib-0130] Wan, M. L. Y. , Ling, K. H. , El‐Nezami, H. , & Wang, M. F. (2019). Influence of functional food components on gut health. Critical Reviews in Food Science and Nutrition, 59(12), 1927–1936. 10.1080/10408398.2018.1433629 29381385

[jfds17487-bib-0131] Wang, G. , Chen, Y. , Xia, Y. , Song, X. , & Ai, L. (2022). Characteristics of probiotic preparations and their applications. Foods, 11(16), 2472. 10.3390/foods11162472 36010472 PMC9407510

[jfds17487-bib-0132] Wang, W. , Rao, L. , Wu, X. , Wang, Y. , Zhao, L. , & Liao, X. (2021). Supercritical carbon dioxide applications in food processing. Food Engineering Reviews, 13, 570–591. 10.1007/s12393-020-09270-9

[jfds17487-bib-0133] Welti‐Chanes, J. , Morales‐de la Peña, M. , Jacobo‐Velázquez, D. A. , & Martín‐Belloso, O. (2017). Opportunities and challenges of ultrasound for food processing: An industry point of view. Ultrasound: Advances for Food Processing and Preservation, 457–497. 10.1016/B978-0-12-804581-7.00019-1

[jfds17487-bib-0134] WIPO . (2019). WIPO green strategic plan 2019–2023: Accelerating the transition to a greener global economy . WIPO at: https://www.wipo.int/edocs/pubdocs/en/wipo_pub_greenstrpl1923.pdf Accessed 10 May 2024

[jfds17487-bib-0135] Xu, M. , Ran, L. , Chen, N. , Fan, X. , Ren, D. , & Yi, L. (2019). Polarity‐dependent extraction of flavonoids from citrus peel waste using a tailor‐made deep eutectic solvent. Food Chemistry, 297, 124970. 10.1016/j.foodchem.2019.124970 31253303

[jfds17487-bib-0136] Yegin, S. , Kopec, A. , Kitts, D. D. , & Zawistowski, J. (2020). Chapter 24 ‐ Dietary fiber: A functional food ingredient with physiological benefits. In H. G. Preuss & D. Bagchi (Eds.), Dietary sugar, salt and fat in human health (pp. 531–555). Academic Press. 10.1016/B978-0-12-816918-6.00024-X

[jfds17487-bib-0137] Yeoh, S. , Shi, J. , & Langrish, T. A. G. (2008). Comparisons between different techniques for water‐based extraction of pectin from orange peels. Desalination, 218(1), 229–237. 10.1016/j.desal.2007.02.018

